# ExosomePurity: tumour purity deconvolution in serum exosomes based on miRNA signatures

**DOI:** 10.1093/bib/bbad119

**Published:** 2023-03-24

**Authors:** Tao Wu, Yao Dai, Yue Xu, Jie Zheng, Shuting Chen, Yinuo Zhang, Peng Tian, Xiaoqi Zheng, Haiyun Wang

**Affiliations:** School of Life Sciences and Technology, Tongji University, Shanghai 200092, China; School of Life Sciences and Technology, Tongji University, Shanghai 200092, China; Shanghai Institute of Hematology, State Key Laboratory of Medical Genomics, National Research Center for Translational Medicine at Shanghai, Ruijin Hospital，Shanghai Jiao Tong University School of Medicine, Shanghai, China; School of Life Sciences and Technology, Tongji University, Shanghai 200092, China; School of Life Sciences and Technology, Tongji University, Shanghai 200092, China; School of Life Sciences and Technology, Tongji University, Shanghai 200092, China; School of Life Sciences and Technology, Tongji University, Shanghai 200092, China; School of Life Sciences and Technology, Tongji University, Shanghai 200092, China; Center for Single-Cell Omics, School of Public Health, Shanghai Jiao Tong University School of Medicine, Shanghai, China; School of Life Sciences and Technology, Tongji University, Shanghai 200092, China

**Keywords:** ExosomePurity, tumour purity, tumour exosomes, miRNA-Seq, early diagnosis

## Abstract

Exosomes cargo tumour-characterized biomolecules secreted from cancer cells and play a pivotal role in tumorigenesis and cancer progression, thus providing their potential for non-invasive cancer monitoring. Since cancer cell-derived exosomes are often mixed with those from healthy cells in liquid biopsy of tumour patients, accurately measuring the purity of tumour cell-derived exosomes is not only critical for the early detection but also essential for unbiased identification of diagnosis biomarkers. Here, we propose ‘ExosomePurity’, a tumour purity deconvolution model to estimate tumour purity in serum exosomes of cancer patients based on microribonucleic acid (miRNA)-Seq data. We first identify the differently expressed miRNAs as signature to distinguish cancer cell- from healthy cell-derived exosomes. Then, the deconvolution model was developed to estimate the proportions of cancer exosomes and normal exosomes in serum. The purity predicted by the model shows high correlation with actual purity in simulated data and actual data. Moreover, the model is robust under the different levels of noise background. The tumour purity was also used to correct differential expressed gene analysis. ExosomePurity empowers the research community to study non-invasive early diagnosis and to track cancer progression in cancers more efficiently. It is implemented in R and is freely available from GitHub (https://github.com/WangHYLab/ExosomePurity).

## INTRODUCTION

Cancer is a genetic disease in which tumour cells grow uncontrolled and invade nearby tissues or spread to other parts of the body [1]. As a leading cause of death worldwide, lung cancer leads to over a million deaths and breast cancer was the most common cancer among women [2–4]. However, cancer mortality can be reduced if patients are diagnosed and treated early. A non-invasive early detection of cancer is of crucial importance for cancer treatment.

Exosomes are a class of extracellular vesicles, which are derived from cells through exocytosis and ingested by target cells, transferring biological signals to local or distant cells [5, 6]. Exosomes contain biomolecules, such as ribonucleic acids (RNAs), microRNAs (miRNAs), deoxyribonucleic acid (DNA), proteins or lipids and are involved in various physiological and pathological processes through autocrine and paracrine signalling [7–12]. To date, the role of exosomes in tumorigenesis and cancer progression is well characterized. For example, in colon cancer, exosomal a disintegrin and metalloproteinase domain 17 (ADAM17) derived from cancer cells facilitated metastasis by cleaving E-cadherin junctions and contributing to the formation of premetastatic niches [13]. Glioma cells have been found to promote M2 polarization of macrophages through the secretion of exosomal miR-3591-3p [14]. Exosomes derived from pancreatic ductal adenocarcinoma (PDAC) were shown to transport CD44v6/C1QBP complexes to the plasma membrane of hepatic satellite cells, promoting hepatic metastasis of PDAC [15]. Transfer of unshielded RN7SL1 in exosomes to breast cancer cells promotes both tumour growth and metastasis [16]. Exosomes can be separated into two discernible subpopulations by AF4 technology and enrich highly heterogeneity biomolecules in various exosomes [17, 18]. Thus, exosomes can be harnessed as an ideal non-invasive diagnosis biomarker [19–21].

It is noteworthy that liquid biopsy of tumour patients contains the mixed sources of exosomes including secreted from cancer cells and healthy cells [22–24]. Identification of cancer cell-derived exosomes from the mixed ones is not only critical for the early detection of cancers but also essential for unbiased identification of diagnosis biomarkers. Therefore, accurately measuring the purity of tumour cell-derived exosomes in liquid biopsy is an efficient approach to address this problem.

The deconvolution method is a common technique in signal and image processing. In these fields, deconvolution is used to reverse the effects of convolution, which is the mathematical operation that occurs when a signal or image is passed through a system that modifies it. By applying deconvolution, one can attempt to recover the original signal or image before it was convolved. In recent years, the deconvolution method has been applied in biology to estimate cellular composition from the methylation data [25, 26], bulk RNA-Seq data [27–30] and spatial transcriptomic data [31]. For example, in the context of bulk RNA-Seq data, CIBERSORT characterizes cell composition of complex tissues from their gene expression profiles using a deconvolution method [29]. TIMER imputes the tumour-infiltrating immune cells from the tumour tissue expression profiles [28]. MethylPurify infers tumour purity using differentially methylated regions from tumour methylome samples [26]. Although these methods yield the satisfactory prediction performance in the purity analysis, there is still lack of the methods of estimating the tumour purity from serum exosomes. An accurate estimation of the purity of cancer cell-derived exosomes from liquid biopsy will make sense to tumour early diagnosis and track cancer progression.

Here we propose ‘ExosomePurity’, a tumour exosome purity deconvolution model to estimate tumour sourced exosome purity in serum exosomes of cancer patients based on miRNA signatures. Firstly, we interrogated miRNA-Seq data to identify the differently expressed miRNAs as miRNA signatures to distinguish cancer cell- from healthy cell-derived exosomes. The generalization of the signatures was evaluated in the independent data. Then the deconvolution model was developed to estimate the tumour exosome purity in serum exosomes of cancer patients. The performance and robustness of this purity model were evaluated on actual and simulated data. Finally, we used the tumour exosome purity to correct differential expressed gene (DEG) analysis.

## MATERIALS AND METHODS

### The framework of tumour purity deconvolution model

**Figure 1:**
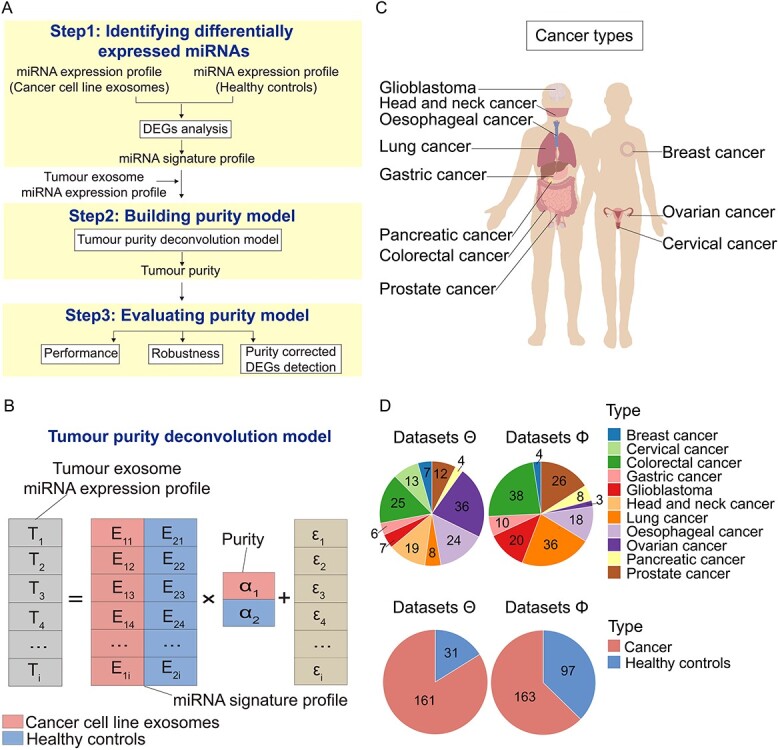
Overview of a tumour purity deconvolution model ‘ExosomePurity’ and the distribution of cancer types used in the model. (**A**) As input for a tumour purity deconvolution model ‘ExosomePurity’, an miRNA signature profile is comprised of miRNAs that are differentially expressed between cancer cell line-derived exosomes and healthy cell-derived exosomes and are stably expressed within each group (Step 1). Given the miRNA signature profile and tumour exosome miRNA expression profile, tumour purity is solved by the purity deconvolution model, which uses quadratic programming to estimate parameters (Step 2). The performance and robustness of the model are evaluated using independent and external samples alone or in combination with noise background. Tumour purity is applied to correct the differentially expressed analysis between tumour exosomes and healthy controls (Step 3). (**B**) Tumour purity deconvolution model. T represents the serum exosome miRNA expression profile of cancer patients. E is the miRNA signature profile of cancer cell line-derived and healthy cell-derived exosomes. α is the proportion matrix of cancer cell- and healthy cell-derived exosomes. (**C**) Schematic of 11 cancer types. Cancer cohorts include breast cancer, cervical cancer, colorectal cancer, gastric cancer, glioblastoma, head and neck cancer, lung cancer, oesophageal cancer, ovarian cancer, pancreatic cancer and prostate cancer. (**D**) Pie chart shows the distribution of 11 cancer types and the number of healthy controls and cancer exosome samples in miRNA-Seq datasets Θ (left) and Φ (right) used in the model.

We developed ‘ExosomePurity’, a tumour purity deconvolution model to estimate the tumour exosome purity in serum exosomes of cancer patients (Figure 1A). Firstly (Step 1), we performed the DEG analysis between cancer cell line-derived exosomes and healthy cell-derived exosomes using miRNA-Seq data. Those miRNAs that are differentially expressed between groups and stably expressed within groups constitute an miRNA signature. We supposed that the expression profile of the miRNA signature represented the miRNA expression pattern of the exosomes secreted from cancer cells in tumour tissue and normal cells. Therefore, the miRNA signature profile can be used to divide the mixed serum exosomes of cancer patients into cancer cell- and healthy cell-derived ones. With this signature profile and exosome miRNA expression profile of cancer patients as input, we built the tumour purity deconvolution model to quantify the proportions of cancer exosomes and normal exosomes in serum (Step 2). Under the assumption that serum exosomes of cancer patients contain two major components of exosomes, from cancer cells and healthy cells, the deconvolution model is formularized as $T= E\alpha +\varepsilon$ (Figure 1B). T represents the serum exosome miRNA expression profile of cancer patients. E is the miRNA signature profile of cancer cell line-derived and healthy cell-derived exosomes. $\alpha$ is the proportion matrix of cancer cell- and healthy cell-derived exosomes. To deconvolve the mixture, we employed the constraint quadratic programming algorithm (see Materials and Methods for detail). We subsequently evaluated the model performance using simulated data mixed by cancer cell- and healthy cell-derived exosomes with a series of different purities as well as actual data (Step 3). The evaluation datasets cover 11 cancer types and include two miRNA-Seq datasets Θ and Φ (Figure 1C–D). The model robustness was evaluated by adding different levels of noise to simulated data. Finally, we utilized the exosome purity calculated by the model to correct differentially expressed miRNAs.

### Tumour purity deconvolution model

We assumed that the serum exosomes of tumour patients are sourced from cancer cell- and healthy cell-derived exosomes, whose miRNA expression patterns can be speculated from cancer cell line exosomes and healthy controls. Here we first generated an miRNA signature profile to depict the expression patterns of miRNAs from cancer cell- and healthy cell-derived exosomes. An miRNA signature profile is expected to use for accurately distinguishing cancer exosomes from healthy controls. We selected the miRNAs that are differentially expressed between cancer cell line exosomes and healthy controls measured by DESeq2 (version 1.30.1) [32] and stably expressed in each subset measured by the variance, to make an miRNA signature. In the present study, miRNAs with |log2FC| > 1 and false discovery rate (FDR) < 0.01 and with variance <2 were considered to be differentially expressed and to be stably expressed, respectively.

In an miRNA signature profile, for any miRNA $i$ in sample $j$, ${E}_{ki}$ represented the expression value of miRNA $i$, which was calculated by the average expression of $i$ in the samples from exosome source k (k = 1 for cancer cell-derived exosomes and k = 2 for healthy cell-derived exosomes) (Equation (1)). For any sample $j$, ${\alpha}_j$ consisted of tumour purity ${\alpha}_{1j}$ and healthy purity ${\alpha}_{2j}$ (Equation (2)).


(1) \begin{equation*} \left(\forall\, \mathrm{i}\in \left[1,\mathrm{m}\right],\forall \mathrm{j}\in \left[1,\mathrm{n}\right]\right):{\mathrm{E}}_{\mathrm{i}}={\left[{\mathrm{E}}_{1\mathrm{i}}{\mathrm{E}}_{2\mathrm{i}}\right]}_{\mathrm{i}=1\to \mathrm{m}} \end{equation*}

(2) \begin{equation*} {\alpha}_j={\left[{\alpha}_{1j}{\alpha}_{2j}\right]}_{j=1\to n} \end{equation*}

For miRNA $i$ in serum exosomes of the patient $j$, we defined the expression level ${t}_{ij}$ was composed of the expression of cancer cell-derived exosomes and healthy cell-derived exosomes (Equation (3)). An array formed by the expression level ${t}_{ij}$, ${T}_{ij}$, is the product of expression value ${E}_j$ and purity array ${\alpha}_j$ (Equations (4) and (5)).


(3) \begin{equation*} {\mathrm{Eki}}\cdot\left(\forall\, \mathrm{i}\in \left[1,\mathrm{m}\right],\forall\, \mathrm{j}\in \left[1,\mathrm{n}\right]\right){:}\,{\mathrm{t}}_{\mathrm{i}\mathrm{j}}={\mathrm{\alpha}}_{1\mathrm{i}}{\mathrm{E}}_{\mathrm{i}1}+{\mathrm{\alpha}}_{2\mathrm{i}}{\mathrm{E}}_{\mathrm{i}2}+\mathrm{\varepsilon} \end{equation*}

(4) \begin{equation*} {T}_{ij}=\left({t}_{1j},{t}_{2j},\dots{t}_{mj}\right)={\left({t}_{ij}\right)}_{i=1\to m} \end{equation*}

(5) \begin{equation*} {T}_{ij}={\alpha}_j{E}_i+\varepsilon \end{equation*}

For each sample $j$, the purity ${\alpha}_j$ should be greater than or equal to 0, and the purity sum of cancer cell-derived exosomes and healthy cell-derived exosomes should be 1 (Equation (6)). The problem is solved by quadratic programming. The solution with the smallest squares of errors is the purity of the sample (Equation (7)). We used the *Solve.QP* function in the quadprog package in R to solve the matrix.


(6) \begin{equation*} s.t.\ \alpha \ge 0,\sum_j^k\alpha =1;\forall j,k,{a}_{jk}\ge 0 \end{equation*}

(7) \begin{equation*} \mathit{\min}{\left\Vert \left(\alpha E-T\right)\right\Vert}^2 \end{equation*}

### Datasets

1) Simulated exosome miRNA-Seq data

We generated simulated miRNA-Seq data of known tumour purity by combining the actual data Θ from cancer cell line exosomes and healthy controls. Simulated tumour exosome data with purity x% were designed by x% expression profile of cancer cell line and (100-x)% of healthy controls. Two purity ranges of datasets were simulated: (1) from 0 to 1 and (2) from 0 to 0.1.

2) Actual exosome miRNA-Seq data

The actual exosome miRNA-Seq data (data Θ, Supplementary Table S1) were obtained from the NCBI Gene Expression Omnibus (GEO; https://www.ncbi.nlm.nih.gov/geo/) [33]. These data included 161 exosome samples of breast cancer, cervical cancer, colorectal cancer, gastric cancer, glioblastoma, head and neck cancer, lung cancer, oesophageal cancer, ovarian cancer, pancreatic cancer and prostate cancer cell lines and 31 healthy control samples, which were used to generate the miRNA signature profile and evaluate the model. In addition, an external cohort (data Φ, Supplementary Table S2) of 163 serum exosome samples of patients with 9 cancers and 97 healthy individuals was also collected, to evaluate the model generalization and detect the differentially expressed miRNAs corrected by tumour purity (Figure 1C-D).

### Tumour-specific miRNA databases

To investigate if miRNAs in the model are tumour specific, we conducted an analysis using three publicly available databases: dbMEMC [34], CancerMIRNome [35] and miRCancer [36]. These databases contain differentially expressed miRNAs in tumour tissues or extracellular fluids of various human cancers. Specifically, dbMEMC and CancerMIRNome collected differentially expressed miRNAs from high-throughput miRNA expression profiles in public data repositories including The Cancer Genome Atlas (TCGA), GEO, Sequence Read Archive and ArrayExpress; miRCancer collected ones by text mining from published literatures.

### Processing of raw miRNA-Seq data

Raw reads of the miRNA-Seq data were processed by removing low-quality reads, adaptor dimers and sequences with lengths < 18 and > 35 nucleotides using cutadapt (version 2.3) (https://cutadapt.readthedocs.io/en/stable/). The filtered reads were aligned to the human genome using bowtie (version 1.2.1) with options ‘-n 1 -l 16 -p 7 -a --best –strata’ [37] and quantified by featureCounts (version 1.5.3) [38], and miRNA annotations were retrieved from miRBase (v22.1) [39]. Expression levels were depicted as counts per million for miRNA. For differential gene expression analysis, raw reads for miRNAs quantified by featureCounts (version 1.5.3) were then analyzed by the Bioconductor package DESeq2 (version 1.30.1) [32].

### Evaluation of performance in simulated and actual exosome miRNA-Seq data

We divided the samples in Θ into five parts, with one of them (Θ_0_) left as an independent cohort and the remaining four parts (Θ_1_) used for 3-fold cross-validation. Additionally, we used external Φ as another independent cohort. More specifically, for simulated data evaluation, we applied two-thirds of Θ_1_ to generate the signatures and the remaining one-third and Θ_0_ to produce simulated data with the varied purities for validation. The purities ranged from 0 to 1 and from 0 to 0.1. The purity 0–0.1 was designed to evaluate if the model works for the early diagnosis of tumour. The Pearson correlation (PC) as a performance benchmarking was applied to evaluate the consistency of the simulated tumour purity and the predicted tumour purity. For actual data evaluation, we applied Θ_1_ for 3-fold cross-validation, and Θ_0_ and Φ as independent cohorts.

To accurately differentiate between tumour and normal samples, we used healthy cell-derived exosomes to generate null distribution (H_0_) of tumour purities, and tested whether the tumour purity (tPurity) for a given sample was from H_0_. If p(tPurity| H_0_) < 0.05, we rejected H_0_ and considered the sample as cancer cell-derived exosomes.

Based on the predictions of samples from cancer cell line-derived exosomes in datasets Θ, patients and healthy individuals in datasets Φ, we used Precision, Recall, Specificity and F1—a combined measure of Precision and Recall—as performance benchmarks to evaluate the model.

### Evaluation of robustness with added noise

We evaluated the robustness of the purity model using simulated exosome miRNA-Seq data with known tumour purity by adding the different levels of noise. The noise follows the Gaussian distributions with mean 0 and SD $\sigma$ 1, 3, 5, 7, 9.

### Detection of differentially expressed miRNAs corrected by tumour purity

For miRNA $i$, we assumed that the expression level ${X}_i\sim N\left({m}_i,{\sigma}_i^2\right)$ in healthy cell-derived exosomes and the expression level ${Y}_i$ were composed of ${X}_i$ and the difference ${\delta}_i$ between cancer cell- and healthy cell-derived exosomes, where ${\delta}_i$ is also assumed to follow normal distribution ${\delta}_i\sim \mathrm{N}\left({\mu}_i,{\tau}_i^2\right)$ (Equation (8)).


(8) \begin{equation*} {Y}_i={X}_{\mathrm{i}}+{\delta}_i \end{equation*}

For miRNA $i$ in serum exosomes of the patient $j$ with purity ${\alpha}_j$, the expression level ${Z}_{ij}$ can be expressed as cancer cell-derived expression levels with proportion ${\alpha}_j$ and healthy exosomes with proportion ($1-{\alpha}_j)$ and ${Z}_{ij}$ follows normal distribution ${Z}_{ij}\sim \mathrm{N}\left({m}_i+{\alpha}_j{\mu}_i,{\varepsilon}_i^2\right)$ (Equations (9) and (10)).


(9) \begin{equation*} {Z}_{\mathrm{ij}}=\left(1-{\alpha}_j\right){X}_{\mathrm{ij}}+{\alpha}_j{Y}_{\mathrm{ij}}={X}_{ij}+{\alpha}_j{\delta}_i \end{equation*}

(10) \begin{equation*} {Z}_{\mathrm{ij}}={m}_i+{\alpha}_j{\mu}_i+\varepsilon \end{equation*}

For ${n}_0$ healthy cell-derived exosomes and ${n}_1$ cancer cell-derived exosomes, $Z$ is the vector of miRNA expression levels, $W$ is the vector of purity of samples, $\beta$ is the parameters determined by the model and $\epsilon$ is the error term (Equation (11)). For given exosome, sequencing data can be described as a linear model (Equation (12)).


(11) \begin{align*} Z&=\left[\kern-12pt\begin{array}{c}{X}_1\\{}{X}_2\\{}\begin{array}{c}\vdots \\{}{X}_{n0}\\{}\begin{array}{c}{Y}_1^{\prime}\\{}{Y_2^{\prime}}_1\\{}\begin{array}{c}\vdots \\{}{Y}_{n1}^{\prime}\end{array}\end{array}\end{array}\end{array}\kern-12pt\right],W=\left[\kern-6pt\begin{array}{c}\begin{array}{c}1\kern0.5em 0\\{}\begin{array}{cc}1& 0\end{array}\\{}\begin{array}{cc}\vdots & \vdots \end{array}\\{}\begin{array}{cc}1& 0\end{array}\\{}\begin{array}{cc}1& {\lambda}_1\end{array}\\{}\begin{array}{cc}1& {\lambda}_2\end{array}\\{}\begin{array}{cc}\vdots & \vdots \end{array}\end{array}\\{}\begin{array}{cc}1& {\lambda}_{n1}\end{array}\end{array}\kern-6pt\right],\beta =\left[\kern-3pt\begin{array}{c}m\\{}\mu \end{array}\kern-3pt\right],\nonumber\\\epsilon &=\left[\kern-12pt\begin{array}{c}{\epsilon}_1\\{}{\epsilon}_2\\{}\begin{array}{c}\vdots \\{}{\epsilon}_{n0}\\{}\begin{array}{c}{\epsilon}_{n0+1}\\{}{\epsilon}_{n0+2}\\{}\begin{array}{c}\vdots \\{}{\epsilon}_{n0+n1}\end{array}\end{array}\end{array}\end{array}\kern-12pt\right] \end{align*}

(12) \begin{equation*} \mathrm{Z}=\mathrm{W}\times \mathrm{\beta} +\varepsilon\qquad\qquad\qquad\qquad\qquad\qquad\qquad\qquad\quad\qquad \end{equation*}

For the hypothesis test ${H}_0:\mu =0$, the Wald test statistics was used to obtain *P*-value. Benjamini–Hochberg’s method is applied on *P*-values to obtain FDRs. The model parameters can be solved by the generalized least square method [40].

In addition, the analysis of uncorrected differentially expressed miRNAs was performed using the Deseq2 packages (version 1.30.1). Differentially expressed miRNAs with FDR values <0.05 and |log2FC| >1 were considered to be significant. Differentially expressed miRNAs were analyzed by DIANA-miRPath (version 3.0) to identify their targets and the Kyoto Encyclopedia of Genes and Genomes (KEGG) signalling pathways [41]. The significance threshold was defined as FDR value <0.05.

### Statistical analysis

The Wilcoxon rank-sum test was used to compare purity. The PC and mean absolute errors (MAE) between predicted and simulated purity were applied to evaluate the performance of the purity model. All statistical analyses were executed in R (version 4.0.3).

## RESULTS

### Identification of miRNA signatures for cancer exosomes

**Figure 2:**
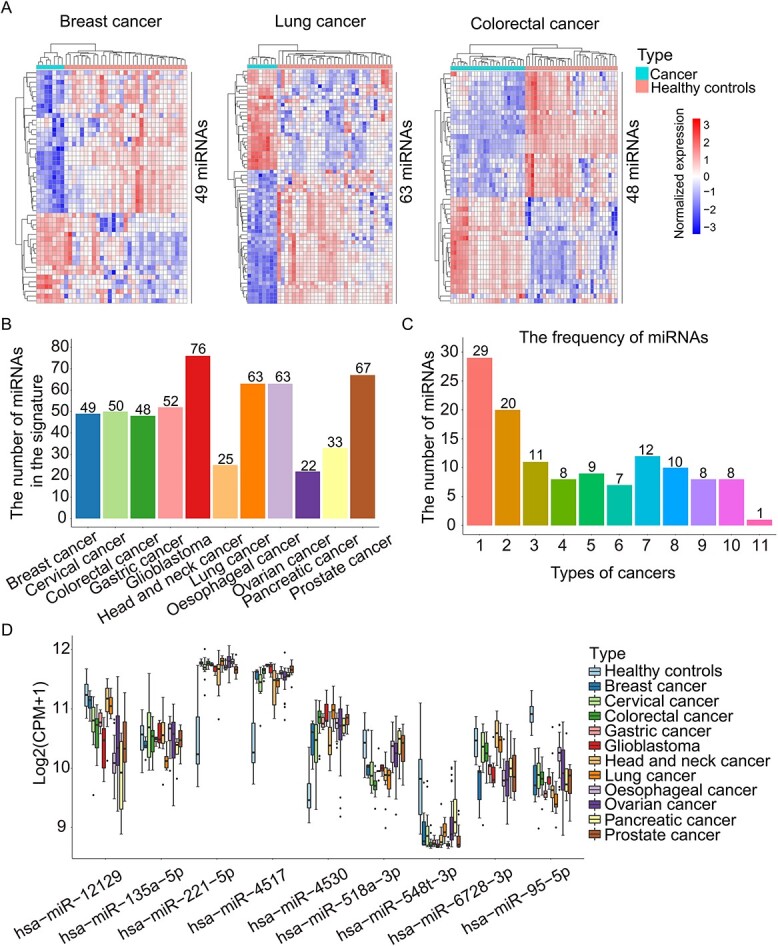
The miRNA signatures of cancer exosomes. (**A**) Heatmap shows the expression levels of miRNA signatures including 49 differentially expressed miRNAs in breast cancer, 63 in lung cancer and 48 in colorectal cancer. Each column represents an exosome sample from cancer or healthy controls. Each row in the heatmap represents a specific miRNA whose expression is normalized across the column. (**B**) The number of miRNAs in the signatures of 11 cancer types (breast cancer, cervical cancer, colorectal cancer, gastric cancer, glioblastoma, head and neck cancer, lung cancer, oesophageal cancer, ovarian cancer, pancreatic cancer and prostate cancer). (**C**) Bar chart shows the frequency of miRNAs in all signatures across 11 cancers. (**D**) Boxplot shows the expression of miRNAs identified in more than nine types of cancers in miRNA-Seq datasets Θ, including hsa − miR − 12129, hsa − miR − 135a − 5p, hsa − miR − 221 − 5p, hsa − miR − 4517, hsa − miR − 4530, hsa − miR − 518a − 3p, hsa − miR − 548 t − 3p, hsa − miR − 6728 − 3p and hsa − miR − 95 − 5p.

To identify miRNA signatures in cancer exosomes, we interrogated miRNA-Seq data Θ and applied Deseq2 to evaluate expression levels across samples. The differentially expressed miRNAs in cancer exosomes were further narrowed down in terms of their expression stability measured by variances with their values less than 2 both in cancer cell- and healthy cell-derived exosomes. Our analysis led to the identification of miRNA signatures in cancer exosomes of 11 cancer types (Figure 2A and Supplementary Figure S1A-H). Especially, we identified 49, 63 and 48 miRNAs, respectively, for breast cancer, lung cancer and colorectal cancer. The average expression of these miRNAs in cancer cell- versus healthy cell-derived exosomes within each cancer constitutes the miRNA signature profile. There were more miRNAs in the signatures of glioblastoma and prostate cancer than of head and neck cancer, pancreatic cancer and ovarian cancer (Figure 2B). Moreover, some miRNAs were identified in multiple types of cancers. For example, there were a total of 46 miRNAs in more than five cancer types (Figure 2C). The miRNAs identified in more than nine cancer types included hsa − miR − 12129, hsa − miR − 135a − 5p, hsa − miR − 221 − 5p, hsa − miR − 4517, hsa − miR − 4530, hsa − miR − 518a − 3p, hsa − miR − 548 t − 3p, hsa − miR − 6728 − 3p and hsa − miR − 95 − 5p, whose expressions were totally different across the different cancers and healthy controls (Figure 2D). Interestingly, hsa − miR − 4530 was identified in all cancers (Supplementary Figure S2A), which was worth further investigation.

To evaluate the robustness of miRNA signatures, we applied re-sampling technology with the two-thirds of samples in datasets Θ to make the signatures and observed the consistency of the signatures when different samples were employed. The results showed that the signatures from three-time sampling were consistent with those from all samples (Supplementary Figure S2B-L). Specially, 79.59% (39), 79.37% (50) and 89.58% (43) of miRNAs in breast cancer, lung cancer and colorectal cancer signatures, respectively, generated by all samples were overlapped with those from sampling.

Our analysis identified 548 miRNAs that are either up- or down-regulated in individual cancers (Supplementary Table S3). We further conducted an analysis using three publicly available databases, dbMEMC, CancerMIRNome and miRCancer, to investigate those miRNAs’ expression in tumour tissues or extracellular fluids of human cancers. Among them, 226 (41.24%) were reported to be up- or down-regulated in at least one database for the same cancer type. If we extended the analysis to include all cancer types without limitations on matching cancer type, we found that 427 out of 548 (77.92%) were up- or down-regulated in at least one database (Supplementary Table S4).

### Evaluation of tumour purity deconvolution model in simulated data

**Figure 3:**
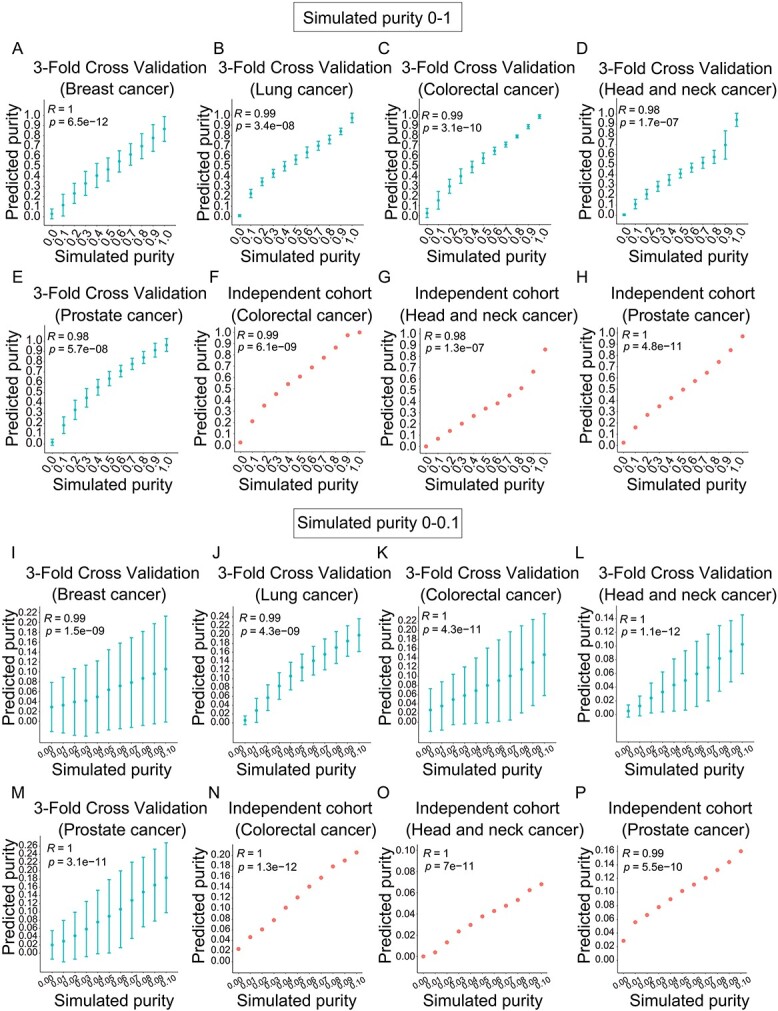
Performance of exosome purity model evaluated in simulated data. The PC between simulated purity and predicted purity for 3-fold cross-validation (**A**-**E**, **I**-**M**) and independent cohort Θ_0_ (**F**-**H**, **N**-**P**). The tumour purity ranges from 0 to 1 (A-H) and from 0 to 0.1 (I-P) in breast cancer (A, I), lung cancer (B, J), colorectal cancer (C, K), head and neck cancer (D, L) and prostate cancer (E, M).

To evaluate whether this model can accurately predict the varied tumour purity, we applied two-thirds of Θ_1_ to generate the signatures and the remaining one-third and Θ_0_ to produce simulated data for validation. The varied purities of the simulated data range from 0 to 1 and from 0 to 0.1 (see section Materials and Methods). The PC was applied to evaluate the consistency of the simulated tumour purity and the predicted tumour purity. The results showed that when the tumour purity ranges from 0 to 1, PCs were 1 in breast cancer, 0.99 in lung cancer and 0.99 in colorectal cancer, showing that the purity model was robust for 3-fold cross-validation (Figure 3A-E and Supplementary Figure S3A-F). Then we generated the simulated exosome miRNA-Seq data based on the independent cohort Θ_0_. The results showed an excellent prediction of this model (Figure 3F-H and Supplementary Figure S3G-I).

To evaluate the ability of this model for early diagnosis, we specially designed the tumour purity varied from 0 to 0.1. We tested the availability of the model by simulation purity ranging from 0 to 0.1 for early diagnosis. We observed that the model presented its extended applicability in mixed exosomes with low tumour purity for 3-fold cross-validation (Figure 3I-M and Supplementary Figure S4A-F). The high correlations between simulated purity and predicted purity were still achieved in the independent cohort Θ_0_ (Figure 3N-P and Supplementary Figure S4G-I). Taken together, the purity model could accurately estimate the purity of simulated exosome data, and miRNA signatures in all cancer types could be generalized to the independent cohort for prediction.

### Evaluation of tumour purity deconvolution model in actual data

We further evaluated the model on two types of actual data: (i) miRNA-Seq datasets Θ and (ii) an external cohort Φ. We divided the samples in Θ into five parts, with one of them (Θ_0_) left as an independent cohort and the remaining four parts (Θ_1_) used for 3-fold cross-validation. Additionally, we used external Φ as another independent cohort.

**Figure 4:**
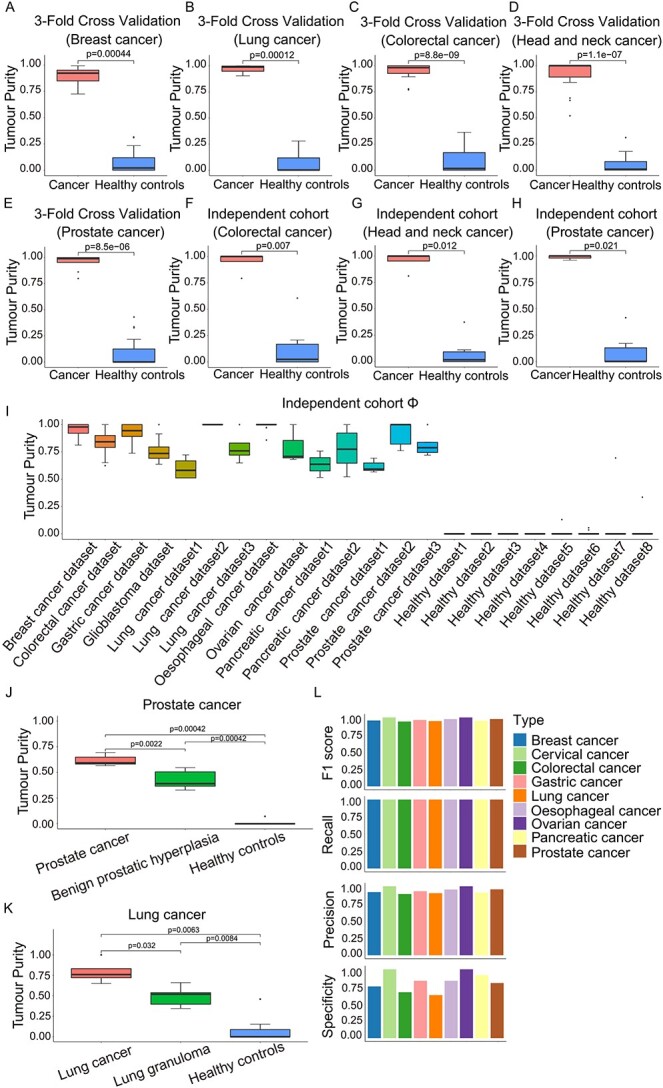
Performance of exosome purity model evaluated in actual data. Comparison of the predicted tumour purity between cancer cell line-derived exosomes (left bars) and healthy controls (right bars) for 3-fold cross-validation (**A**-**E**) in breast cancer (A), lung cancer (B), colorectal cancer (C), head and neck cancer (D) and prostate cancer (E). Comparison of the predicted tumour purity between cancer cell line-derived exosomes (left bars) and healthy controls (right bars) for independent cohort Θ_0_ (**F**-**H**) in colorectal cancer (F), head and neck cancer (G) and prostate cancer (H). The *P* value is calculated with the Wilcoxon rank-sum test. (**I**) The predicted tumour purity of external cohort Φ. The boxplots show the purity of tumour patients (left bars), early disease states (middle bars) and healthy controls (right bars) in prostate cancer (**J**) and lung cancer (**K**). The model performance is evaluated using Precision, Recall, Specificity and F1 in the combined samples in datasets Θ, patients and healthy individuals in datasets Φ (**L**). The *P* value is calculated with the Wilcoxon rank-sum test.

The results demonstrated good ability of our model to distinguish two groups of samples of Θ in 3-fold cross-validation (Figure 4A-E and Supplementary Figure S5A-F) and independent cohort (Figure 4F-H and Supplementary Figure S5G-I). The median of predicted purity was close to 1 for cancer cell-derived exosomes and to 0 for healthy cell-derived exosomes, suggesting the accuracy of the model.

**Figure 5:**
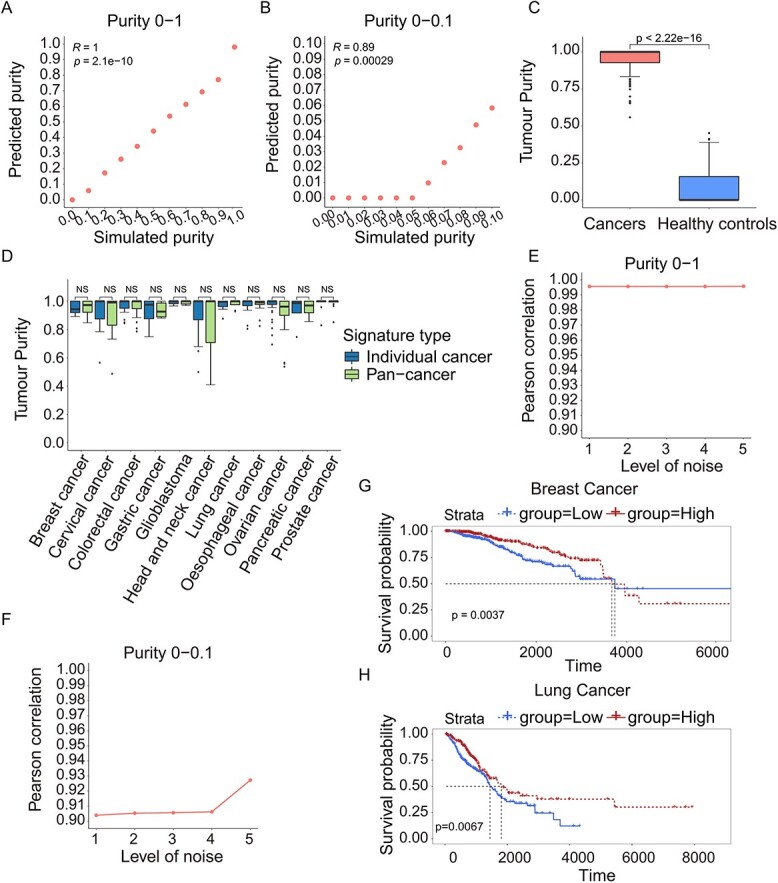
Performance of pan-cancer exosome purity model evaluated in simulated data and actual data. The PC between simulated and predicted exosome purity for all samples with the tumour purity ranging from 0 to 1 (**A**) and from 0 to 0.1 (**B**) in pan-cancer exosome purity model. (**C**) Comparison of the predicted tumour purity between cancer cell line-derived exosomes (left bars) and healthy controls (right bars) for all samples. (**D**) Comparison of the predicted tumour purity with 11 individual cancer signatures and pan-cancer signatures for 11 different cancer cell line-derived samples. The *P* value is calculated with the Wilcoxon rank-sum test. The PC between the simulated and predicted tumour purity ranging from 0 to 1 (**E**) and from 0 to 0.1 (**F**) when the different levels of noise added. The Kaplan–Meier curve illustrates the probability of OS according to the average expression levels of the 28 down-regulated miRNAs in breast cancer (**G**) and lung cancer (**H**).

Furthermore, miRNA signatures were generalized to an external cohort Φ, including exosome datasets from the patients with nine cancer types and healthy controls. The results showed that the purity of different cancer samples varied considerably and healthy controls were close to 0 absolutely (Figure 4I). In particular, we noted that the model distinguished the different disease states well (Figure 4J-K). The median of tumour purity for the patients with prostate cancer was close to 0.6, which was significantly higher than the patients with benign prostatic hyperplasia (*P* = 0.0022, Wilcoxon rank-sum test). Besides, a similar result was observed between the patients with lung cancer and lung granuloma (*P* = 0.032, Wilcoxon rank-sum test).

To accurately differentiate between the exosomes secreted from tumour samples and from normal samples, we used healthy cell-derived exosomes to generate null distribution (H_0_) of tumour purities, and tested whether cancer cell line-derived exosomes in datasets Θ, patients’ and healthy individuals’ exosomes in datasets Φ can be accurately predicted to be from tumour or normal samples. We further used Precision, Recall, Specificity and F1—a combined measure of Precision and Recall—as performance benchmarks to evaluate the model. The results demonstrated our model achieved high performance with zero false negatives and acceptable false positives (Figure 4L).

### Robustness and precision of tumour purity deconvolution model

To evaluate the robustness of the purity model, we added the different levels of noise into the simulated data. The noise follows the Gaussian distributions with mean 0 and SD σ 1, 3, 5, 7, 9. We assessed the model robustness by two measurements, the PC and MAE, between predicted purity and simulated purity. The model was shown to be stable at the different levels of noise. For each cancer with simulated varied purity, the model achieved the PCs above 0.9 and high PCs were maintained when noise levels were increasing (Supplementary Figure S6A-B). We then observed that MAE values overall were very low (Supplementary Figure S6C-F), tending to rise along with the increase of tumour purity (Supplementary Figure S6C-D) and the added noise (Supplementary Figure S6E-F). Therefore, the purity model is robust against the noise. Of note, the model is much robust at low purity, indicating its potential in tumour early diagnosis (Supplementary Figure S6D, F).

### Evaluation of pan-cancer purity deconvolution model in simulated/actual data

Our analysis above generated cancer type-specific model and achieved good performance in predicting tumour purity. Next, since some miRNAs were identified in multiple types of cancers (Figure 2C), we selected 46 miRNAs that were present in more than five types of cancers as pan-cancer miRNA signatures to generate and evaluate the pan-cancer purity deconvolution model. We generated the simulated exosome miRNA-Seq data with the tumour exosome purity ranging from 0 to 1 and from 0 to 0.1 based on miRNA-Seq datasets Θ. The results showed that PCs were high when the tumour exosome purity ranging from 0 to 1 and 0 to 0.1 (Figure 5A and B). The pan-cancer purity model still well distinguished all cancer cell line-derived exosome samples from healthy cell-derived exosome samples in actual data Θ (Figure 5C). We also compared the predicted tumour purity of an individual cancer type using pan-cancer miRNA signatures and individual cancer miRNA signatures. The results showed that there were no significant differences between the miRNA signatures of pan-cancer and individual cancer, indicating that the pan-cancer model can predict tumour purity as well (Figure 5D). Moreover, at the different levels of noise, the pan-cancer model achieved the PCs above 0.9, showing high stability (Figure 5E and F). These results showed that the pan-cancer tumour purity deconvolution model using 46 miRNA signatures achieved excellent prediction performance and was applicable to any individual cancers.

To further understand the function of these 46 miRNAs, including 18 up-regulated and 28 down-regulated ones, we investigated their expression in tumour tissues or extracellular fluids of various human cancers using three publicly available databases, dbMEMC, CancerMIRNome and miRCancer, as well as their predictive ability on overall survival (OS) using TCGA patient data. Notably, 41 (89%) were reported to be up- or down-regulated in at least one database (Supplementary Table S5). Next, TCGA patients were divided into two groups, high-expression and low-expression, according to the average expression levels of the down-regulated or up-regulated miRNAs. Remarkably, 28 down-regulated miRNAs well presented their predictive ability on OS in breast cancer and lung cancer (*P* <0.01, Figure 5G and H).

### Differential analysis corrected by tumour purity

Our analysis showed that tumour purity varied among tumour exosomes, moreover, about 10%–40% of which were from healthy cell-derived ones (Figure 4I). This will lead to biased identification of the differentially expressed miRNAs if tumour purity is not taken into account in differential analysis. We thus developed a method to correct differential analysis using tumour purity, which was applied to serum exosome samples of colorectal cancer, glioblastoma, pancreatic cancer, gastric cancer and lung cancer (see Materials and Methods). After purity correction, there were 71, 190, 46, 36 and 49 differentially expressed miRNAs, respectively, for colorectal cancer, glioblastoma, pancreatic cancer, gastric cancer and prostate cancer (Supplementary Figure S7A-E). Among them, 44, 130, 28, 20 and 21 miRNAs were also identified by Deseq2 analysis without adjusting tumour purity, and 27, 60, 18, 16 and 28 miRNAs were uniquely identified by our method. We performed the KEGG analysis on target genes of the 27, 60, 18, 16 and 28 differentially expressed miRNAs in five cancer types. Interestingly, the mitogen-activated protein kinase (MAPK) signalling pathway and the PI3K-Akt signalling pathway were found to be the top enriched pathways in those cancers (Supplementary Figure S7F-J), which were well consistent with the vital roles of these pathways in cancers. These results suggested that the differentially expressed miRNAs identified after purity correction may provide more biological meanings for further investigation.

## DISCUSSION

Tumour-derived exosomes can be harnessed as non-invasive diagnosis and prognostic biomarkers because they are enriched in biological fluids and carry tumour-characterized biomolecules [8]. Exosomes from liquid biopsy of cancer patients are mixed by tumour cell- and healthy cell-secreted ones. Therefore, accurate and sensitive detection of tumour cell-derived exosomes in biological fluids is an efficient approach for the early diagnosis and tracking of cancer progression. Currently, numerous methods were developed to estimate tissue tumour purity [42–45]. However, there is still a lack of a method to estimate the tumour purity from tumour biological fluids.

Therefore, we propose the R-based tumour purity deconvolution model ‘ExosomePurity’ to address this unmet need and enable researchers to accurately estimate tumour exosome purity from miRNA-Seq data in serum exosomes of cancer patients. Our study currently used this model in 11 cancer types. Utmost, it can be extended to any cancers, provided sufficient serum exosome sequencing data. The purity model was evaluated by actual and simulated data with purity ranging from 0 to 1 and from 0 to 0.1 as the application for early diagnosis. The median predicted purity is close to 1 in actual cancer cell-derived exosomes and close to 0 in actual healthy cell-derived exosomes. The purity predicted by the model shows high correlation with simulated purity in simulated data (Supplementary Figure S8). In addition, the model is robust under the different levels of noise background. Thus, our model gains the good prediction performance for serum tumour exosomes. When applied to simulated data with the varied purities, the model successfully predicted samples with the purity greater than 0.2, indicating its potential for early cancer diagnosis. Moreover, cancer patients at the different cancer progression introduce variations in tumour purity, leading to the biased identification of biomarkers. We further used tumour purity to correct the DEGs. The new DEGs obtained after purity correction are enriched in cancer-related signalling pathways.

In recent years, numerous studies have shown that miRNAs can be circulated in biological fluids and serve as the biomarkers for diagnosis and prognosis. For example, hsa-miR-21 is involved in glioblastoma development and can predict tumour recurrence or metastasis [46]. Moreover, hsa-miR-21 shows a higher upregulation in stage II PDAC and intraductal papillary mucinous neoplasm (IPMN) patients, suggesting that it can thus serve as early diagnostic markers of these two cancers [47, 48]. Hsa-miR-9-5p is identified to be down-regulated in pancreatic cancer by the differential analysis corrected by tumour purity in our analysis. Overexpression of miR-9-5p significantly inhibits proliferation and suppresses the invasion of pancreatic cancer cells [49]. In adenocarcinoma, miR-9-5p exerts a tumour suppressive role and the epithelial-to-mesenchymal transition phenotype is achieved by low levels of miR-9-5p, which enable the upregulation of CDH2 via the transcription factor TWIST1 [50]. Although the differentially expressed miRNAs in 11 cancer exosomes are different due to the regulatory heterogeneity of miRNAs across cancers (Supplementary Table S3), our analysis still identified some miRNAs, which are consistently up-regulated or down-regulated in multiple cancers (Supplementary Table S4 and Supplementary Table S5). For example, hsa-miR-200c-3p is up-regulated in seven types of cancers. Interestingly, it has been reported as a novel biomarker in endometrial cancer patients from a non-invasive liquid biopsy screening of urine-derived exosomes [51]. In addition, as an miRNA up-regulated in two types of cancer, hsa-miR-100 has been reported to play a significant role in cancer progression and is considered as a prognostic biomarker for cancer [52–56]. Also, as a transforming growth factor beta effector, hsa-miR-100 regulates the p53 pathway and DNA repair signalling and apoptosis [53]. Hsa-miR-100 is up-regulated in kirsten rat sarcoma viral oncogene homolog (KRAS) mutant colorectal cancer exosomes and confers hsa-miR-100 mediated cell communication [56]. Moreover, 46 miRNAs (Supplementary Table S3) that were present in more than five types of cancers gain the good prediction ability in the pan-cancer deconvolution model and thus provide the further evidence of exosome miRNAs in tumorigenesis and development. And the mechanisms of their regulatory role in cancers deserve further investigation.

Tumour purity is an important measurement for tumour samples, reflecting cancer progression, tumour microenvironment, the perturbed pathways et al [57]. Our study tentatively measured tumour purity in biological fluids, pushing forward to its application in non-invasive early diagnosis and cancer progression monitor. However, tumour exosomes deliver specific cargo of biomolecules, which is heterogenous between the patients and at the different stages [18]. Moreover, there is limited knowledge of exosome-specific molecular machineries of biogenesis and release. When more serum exosome samples as well as knowledge are available in future, some efforts should definitely include the optimization of the miRNA signature and the rational stratification of samples in the model. Additionally, our model needs to be continuously updated with the emergence of additional sequencing datasets, especially those including tissues of precancerous lesions, to refine the model and improve its ability to detect early cancer.

In summary, we developed ExosomePurity, a tumour exosome purity deconvolution model to estimate tumour sourced exosome purity in serum exosomes of cancer patients based on miRNA signatures. ExosomePurity empowers the research community to study non-invasive early diagnosis and track cancer progression in cancers more efficiently.

**Key Points**

- We propose ‘ExosomePurity’, a tumour purity deconvolution model to estimate tumour purity in serum exosomes of cancer patients based on miRNA signatures.
- The deconvolution models of individual cancers and pan-cancer are developed and gain the excellent performance in simulated and actual data of 11 individual cancers and pan-cancer.
- ExosomePurity generates miRNA signatures of individual cancers and pan-cancer, which achieve the good prediction ability for tumour purity and clinical outcome, deserving further investigation on their regulatory mechanisms during tumorigenesis and development.
- ExosomePurity empowers the research community to study non-invasive early diagnosis and track cancer progression in cancers efficiently.

## Supplementary Material

Supplementary_Figures_bbad119

Table_S1_S2_S3_bbad119

## Data Availability

The datasets generated and/or analyzed during the current study are available in the Gene Expression Omnibus (GEO), [https://www.ncbi.nlm.nih.gov/geo/].

## Notes

**Tao Wu** is a PhD student in the School of Life Sciences and Technology, Tongji University, China. His research focuses on developing statistical methods for the integrative analysis of multi-omics data.

**Yao Dai** is a master student in the School of Life Sciences and Technology, Tongji University, China. Her research focuses on non-coding RNA, bioinformatics, machine learning and multi-omics data mining.

**Yue Xu** is a PhD student in the School of Life Sciences and Technology, Tongji University, China. Her research focuses on developing and applying informatics methods for analyzing epigenetics, omics and other biological data.

**Jie Zheng** is a PhD student in the School of Life Sciences and Technology, Tongji University, China. His research focuses on high-throughput sequencing data analysis, machine learning, bioinformatics and data mining.

**Shuting Chen** is a master student in the School of Life Sciences and Technology, Tongji University, China. Her research focuses on high-throughput sequencing data analysis, deep learning, bioinformatics and drug discovery.

**Yinuo Zhang** is a PhD student in the School of Life Sciences and Technology, Tongji University, China. His research focuses on developing and applying informatics and computing methods for analyzing genomics.

**Peng Tian** is a master student in the School of Life Sciences and Technology, Tongji University, China. His research focuses on complex networks, machine learning, deep learning and drug discovery.

**Xiaoqi Zheng** is a professor in the Center for Single-Cell Omics, School of Public Health, Shanghai Jiao Tong University School of Medicine, China. His research focuses on developing statistical models of the 3D structure of chromatin and computational models in precision medicine and computational biology.

**Haiyun Wang** is a professor in the School of Life Sciences and Technology, Tongji University, China. Her research focuses on tumour heterogeneity, mechanism of drug resistance, drug combination in cancer and novel biomarkers of cancer.
