# Supplementary material for: ExosomePurity: tumour purity deconvolution in serum exosomes based on miRNA signatures
Source: Brief Bioinform. 2023 Mar 24;24(3):bbad119. doi: 10.1093/bib/bbad119 (PMC10199770; doi:10.1093/bib/bbad119)
Supplement: Supplementary_Figures_bbad119 [file supplementary_figures_bbad119.docx]

**Supplementary Figures**

**Supplementary Figure S1**


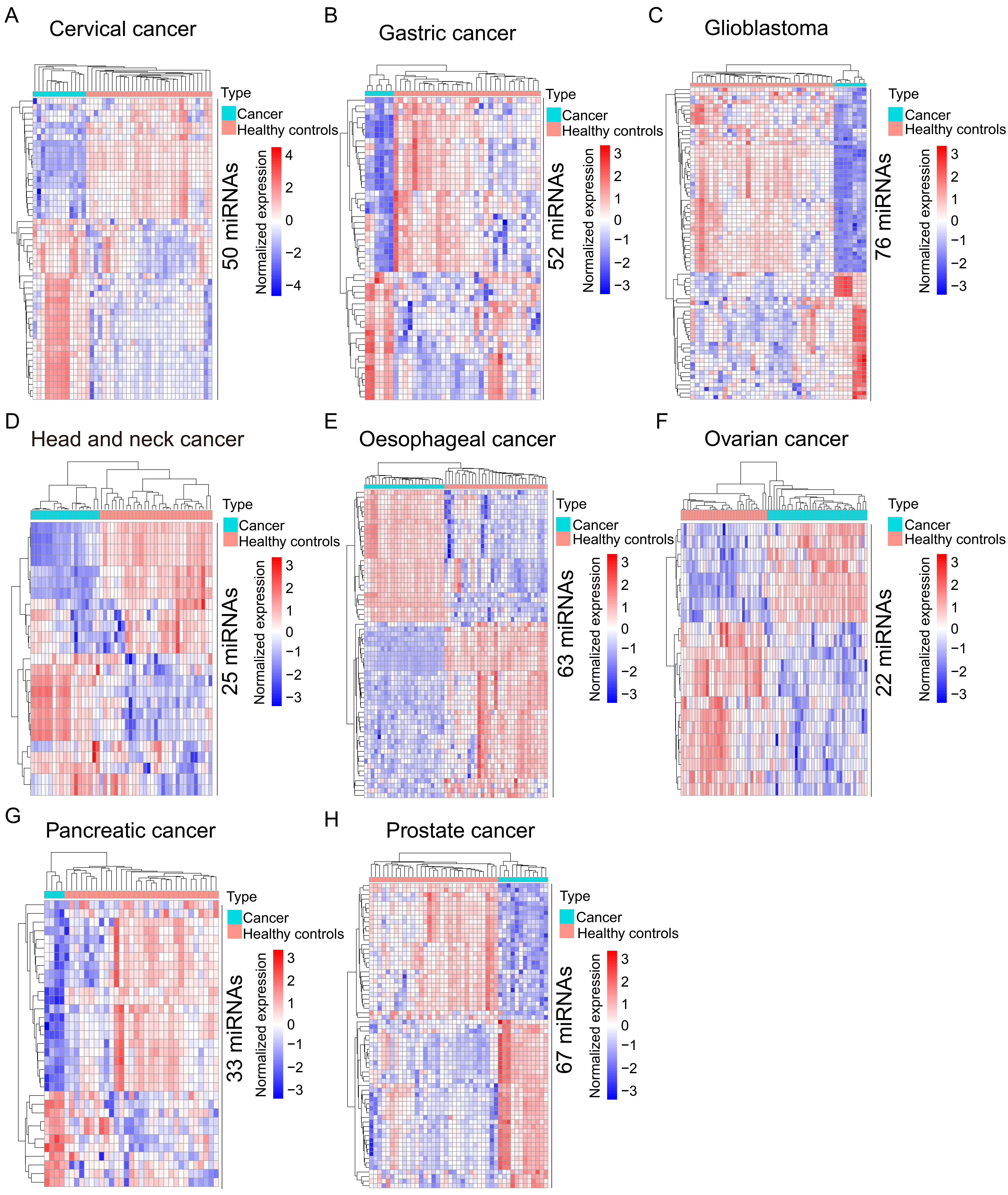


**Supplementary Figure S1. The heatmap of 8 cancer types miRNA signatures.** Heatmap shows the expression levels of miRNA signatures including 50 differentially expressed miRNAs in cervical cancer **(A)**, 52 in gastric cancer **(B)**, 76 in glioblastoma **(C)**, 25 in head and neck cancer **(D)**, 63 in oesophageal cancer **(E)**, 22 in ovarian cancer **(F),** 33 in pancreatic cancer **(G)** and 67 in prostate cancer **(H)**. Each column represents an exosome sample with green for cancer, and red for healthy controls. Each row in the heatmap represents a specific miRNA whose expression is normalized across the column, with high expression shown in red and low expression shown in blue.

**Supplementary Figure S2**

**
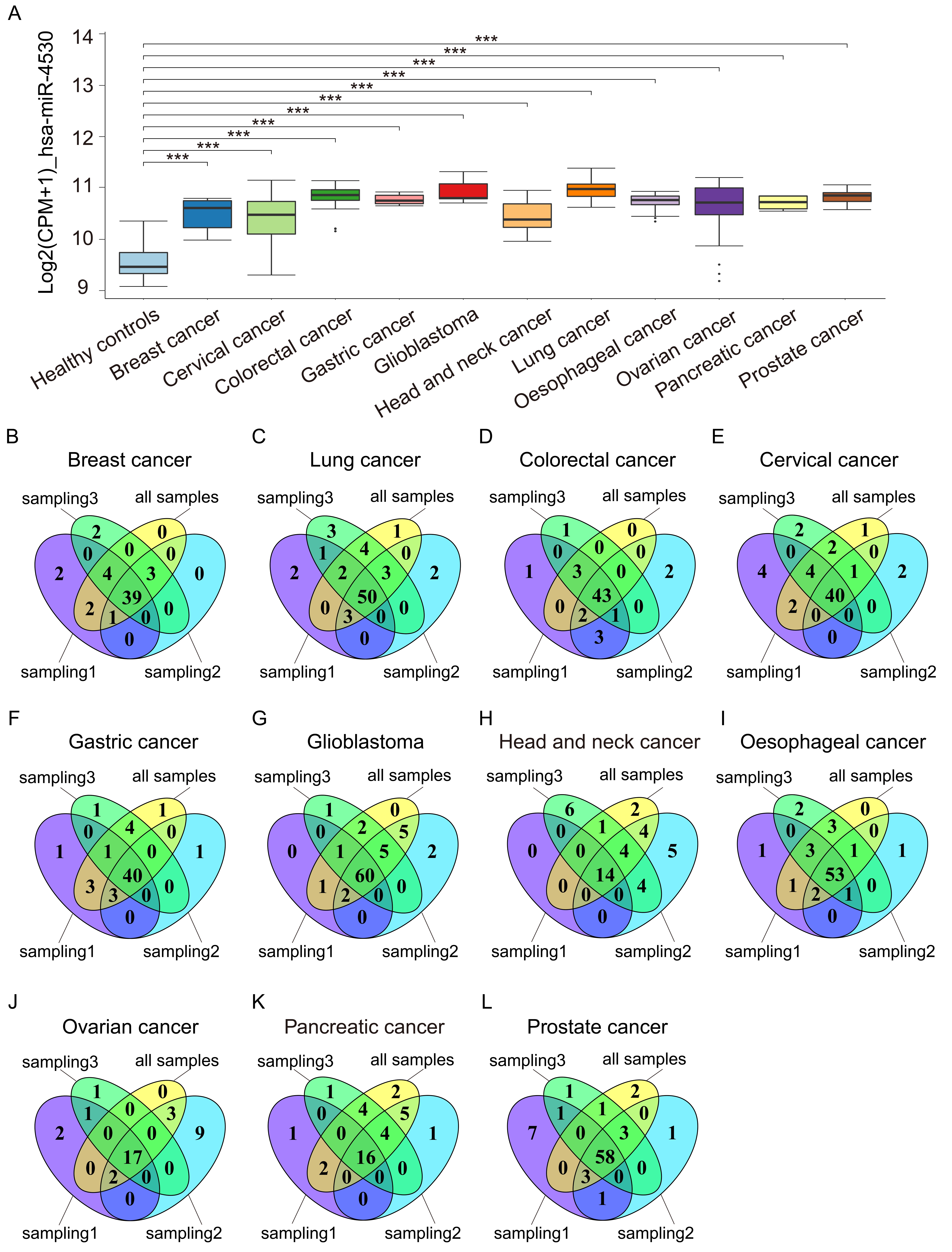
**

**Supplementary Figure S2. Overview of the cancer exosomes miRNA signatures. (A)** Boxplot shows the expression of miRNAs hsa-miR-4530 in miRNA-Seq datasets Θ. The statistical significance between groups is determined by Wilcoxon rank-sum test (*p < 0.05, **p < 0.01, ***p < 0.001, ****p < 0.0001). Overlap of the miRNAs generated by two-thirds of samples and all samples in breast cancer **(B)**, lung cancer **(C)**, colorectal cancer **(D)**, cervical cancer **(E)**, gastric cancer **(F)**, glioblastoma **(G)**, head and neck cancer **(H)**, oesophageal cancer **(I)**, ovarian cancer **(J)**, pancreatic cancer **(K)** and prostate cancer exosomes **(L)**.

**Supplementary Figure S3**


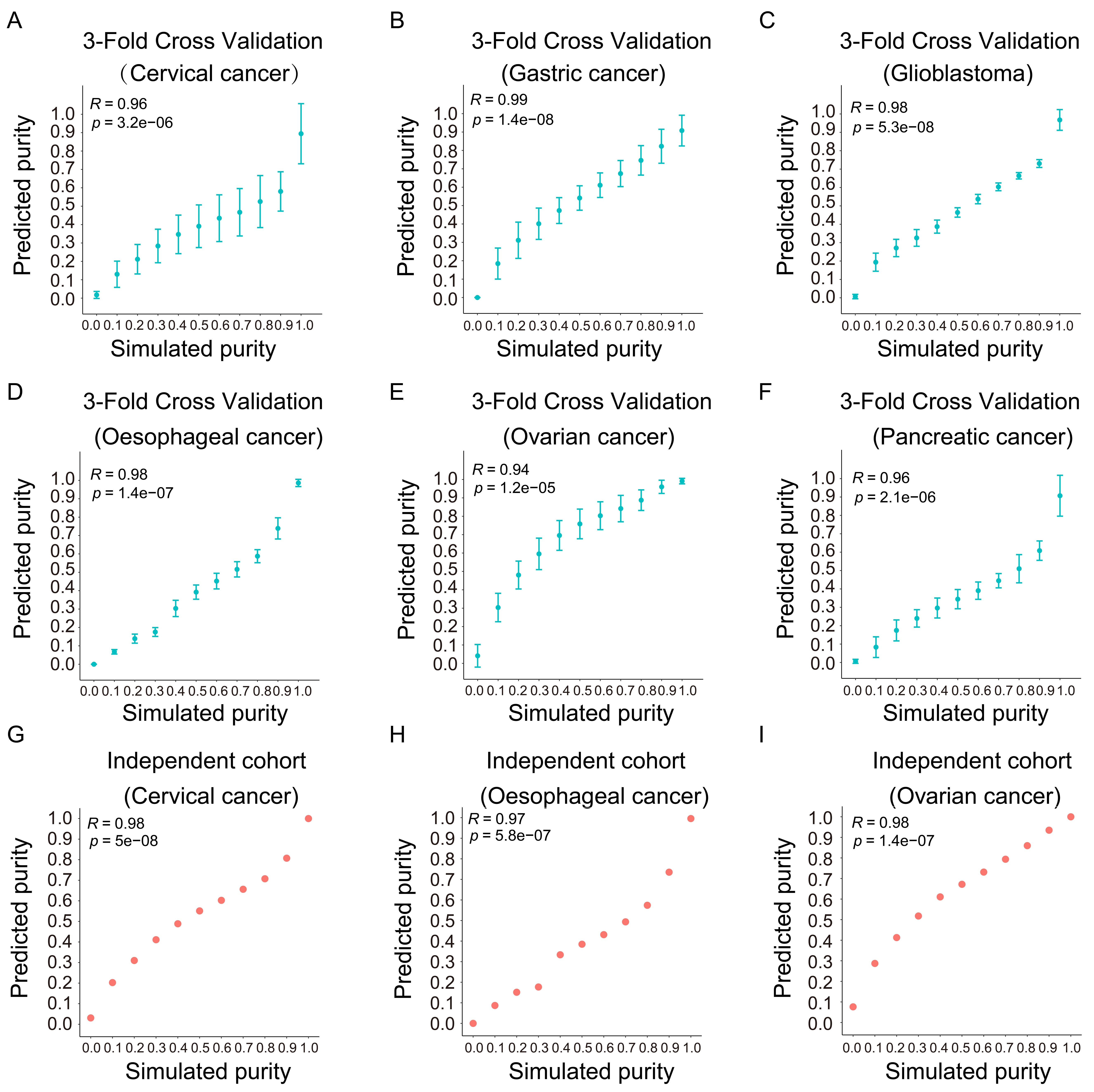


**Supplementary Figure S3. Performance of purity model evaluated in simulated data when using three-fold cross-validation and independent cohort Θ_0_ (purity ranging from 0 to 1).** The pearson correlation between simulated and predicted exosome purity for three -fold cross-validation with the tumour purity ranging from 0 to 1 in cervical cancer **(A)**, gastric cancer **(B)**, glioblastoma **(C)**, oesophageal cancer **(D)**, ovarian cancer **(E)** and pancreatic cancer **(F)**. The pearson correlation between simulated and predicted exosome purity for independent cohort Θ_0_ with the tumour purity ranging from 0 to 1 in cervical cancer **(G),** oesophageal cancer **(H)** and ovarian cancer **(I)**.

**Supplementary Figure S4**


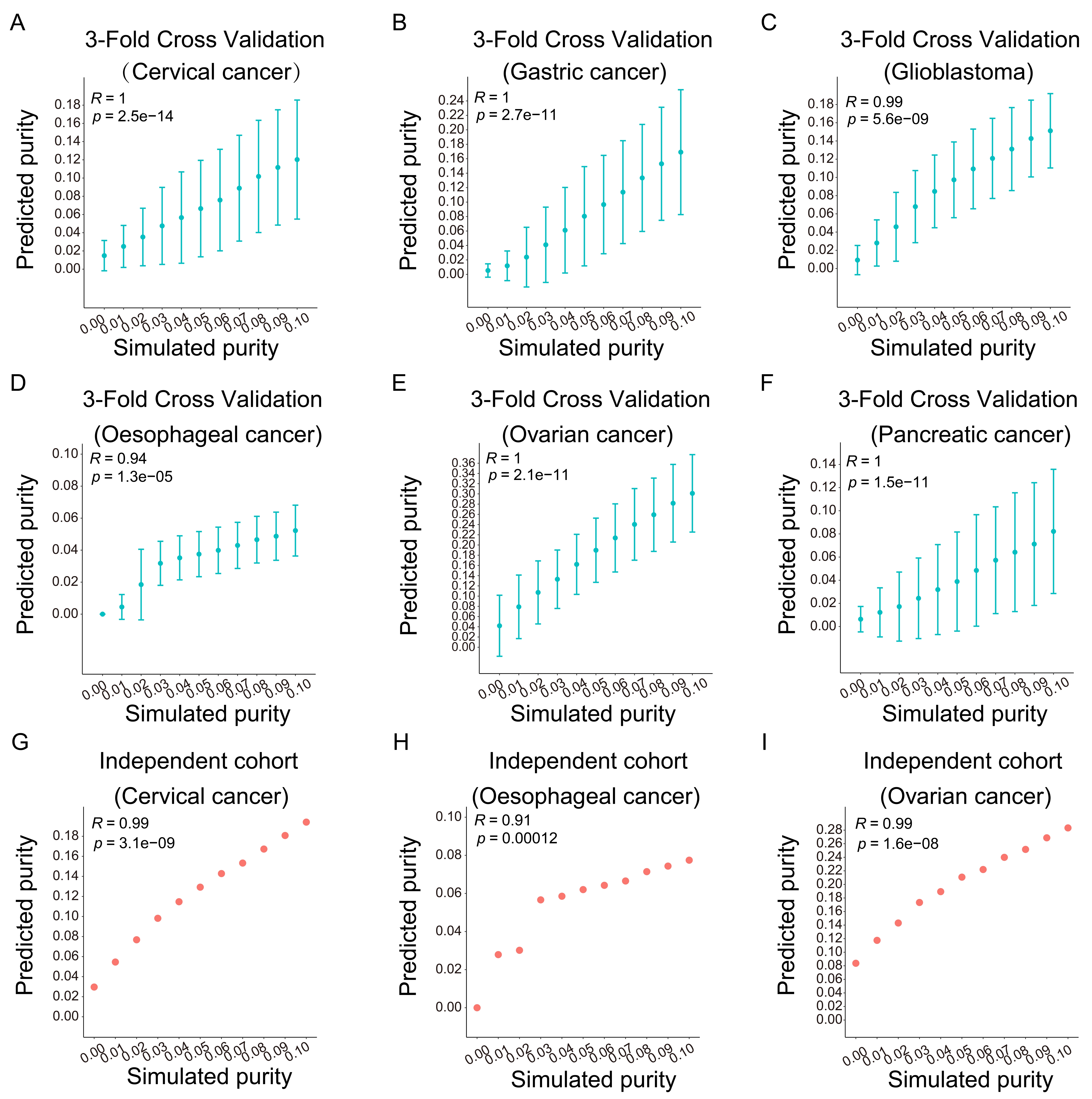


**Supplementary Figure S4. Performance of purity model evaluated in simulated data when using three-fold cross-validation and independent cohort Θ_0_ (purity ranging from 0 to 0.1).** The pearson correlation between simulated and predicted exosome purity for three -fold cross-validation with the tumour purity ranging from 0 to 0.1 in cervical cancer **(A)**, gastric cancer **(B)**, glioblastoma **(C)**, oesophageal cancer **(D)**, ovarian cancer **(E)** and pancreatic cancer **(F)**. The pearson correlation between simulated and predicted exosome purity for independent cohort Θ_0_ with the tumour purity ranging from 0 to 0.1 in cervical cancer **(G),** oesophageal cancer **(H)** and ovarian cancer **(I)**.

**Supplementary Figure S5**


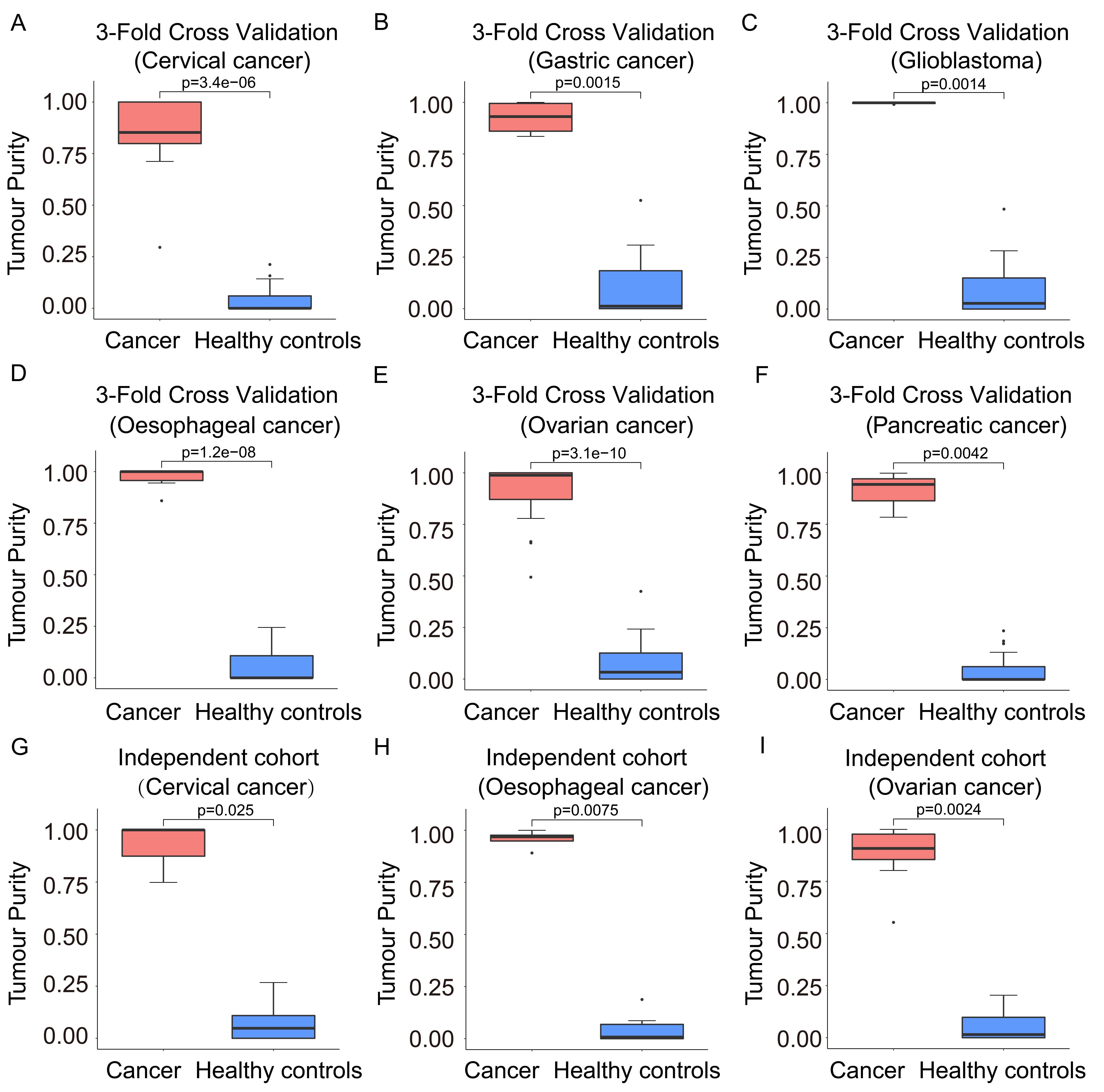


**Supplementary Figure S5. Performance of purity model evaluated in actual data when using three-fold cross-validation and independent cohort Θ_0_.** Comparison of the predicted tumour purity between cancer cell line-derived exosomes (red bars) and healthy controls (blue bars) for three -fold cross-validation in cervical cancer **(A)**, gastric cancer **(B)**, glioblastoma **(C)**, oesophageal cancer **(D)**, ovarian cancer **(E)** and pancreatic cancer **(F).** Comparison of the predicted tumour purity between cancer cell line-derived exosomes (red bars) and healthy controls (blue bars) for independent cohort Θ_0_ in cervical cancer **(G)**, oesophageal cancer **(H)**, ovarian cancer **(I)**. The P value is calculated with the Wilcoxon rank-sum test.

**Supplementary Figure S6**


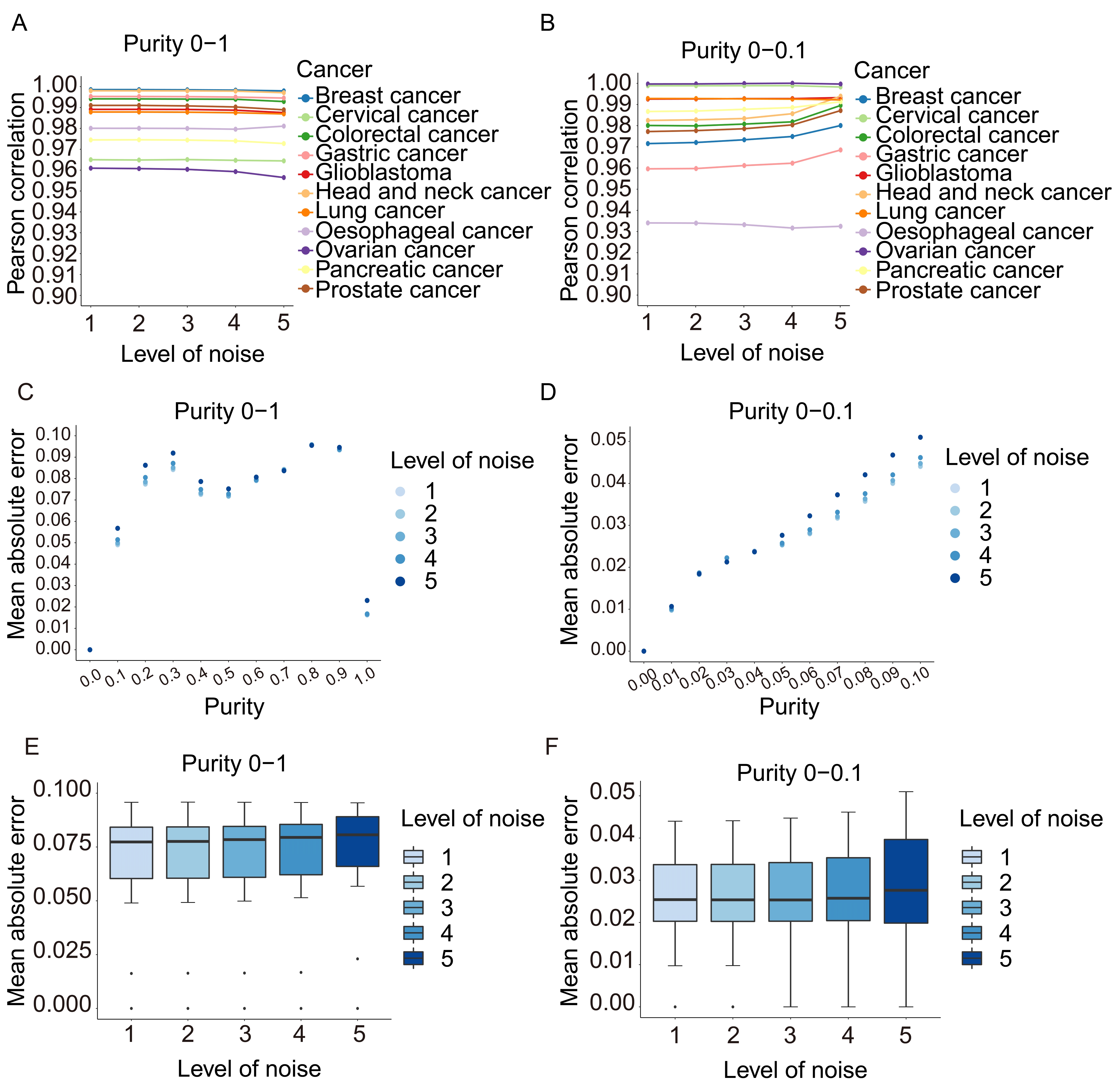


**Supplementary Figure S6. Robustness and precision of exosome purity model.** The robustness of model is evaluated by adding different noise background into simulated tumour exosome expression profile with a series of different tumour purities. The pearson correlation between the simulated and predicted tumour purity ranging from 0 to 1 **(A)** and from 0 to 0.1 **(B)** when the different levels of noise added. Mean absolute errors between the predicted and simulated purity with the tumour purity ranging from 0 to 1 **(C)** and from 0 to 0.1 **(D)**. Mean absolute errors between the predicted and simulated purity ranging from 0 to 1 **(E)** and from 0 to 0.1 **(F)** for the different levels of noise.

**Supplementary Figure S7**

**
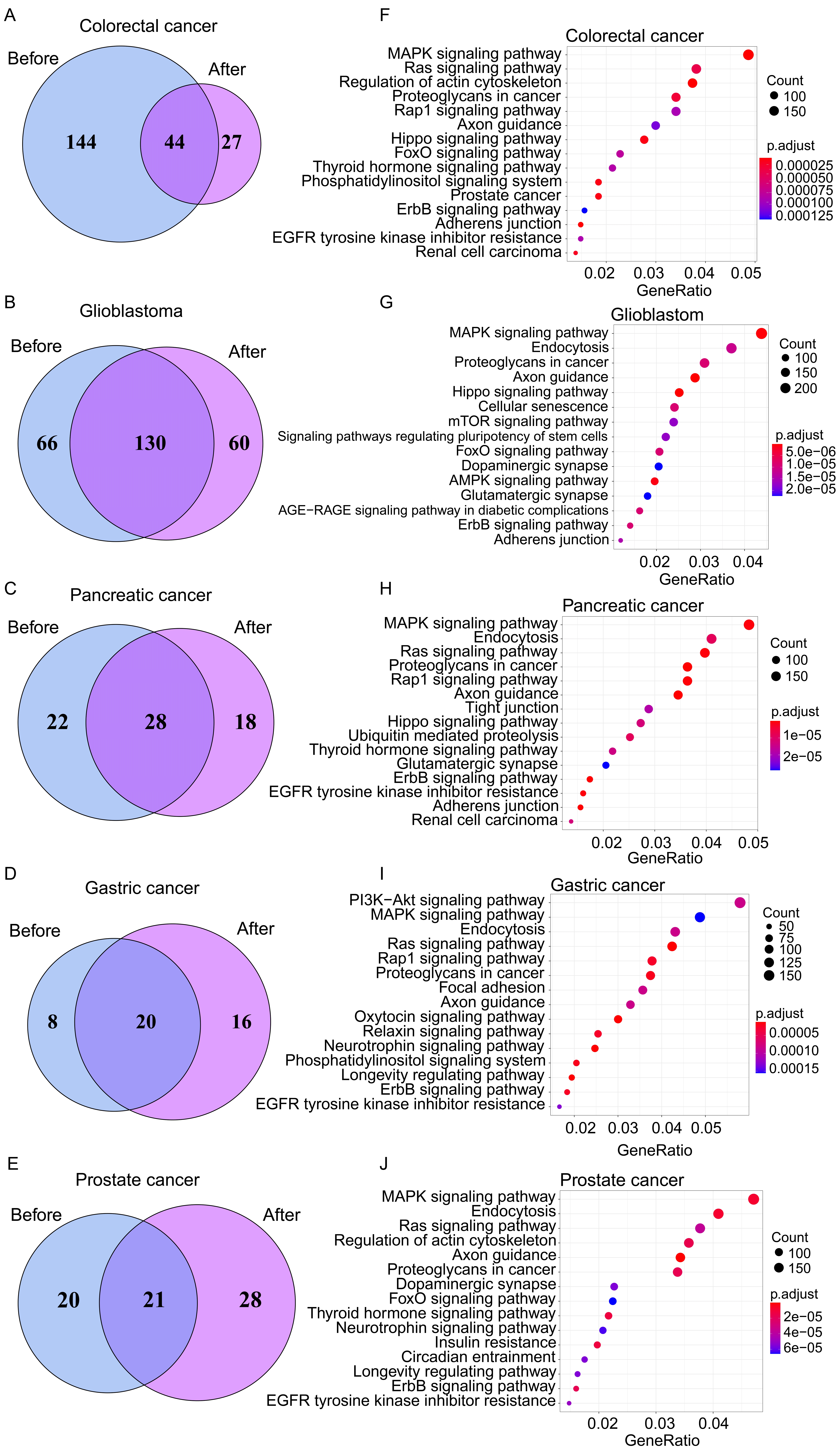
**

**Supplementary Figure S7. The differentially expressed miRNAs in tumour exosomes corrected by tumour purity.** Venn diagrams of differentially expressed miRNAs before and after exosome purity correction in colorectal cancer **(A)**, glioblastoma **(B)**, pancreatic cancer **(C)**, gastric cancer **(D)** and prostate cancer **(E)**. The KEGG pathway enrichment analysis of the targets of 27, 60, 18, 16 and 28 differentially expressed miRNAs that are uniquely identified after purity correction in colorectal cancer **(F)**, glioblastoma **(G)**, pancreatic cancer **(H)**, gastric cancer **(I)** and prostate cancer **(J)**.

**Supplementary Figure S8**

**
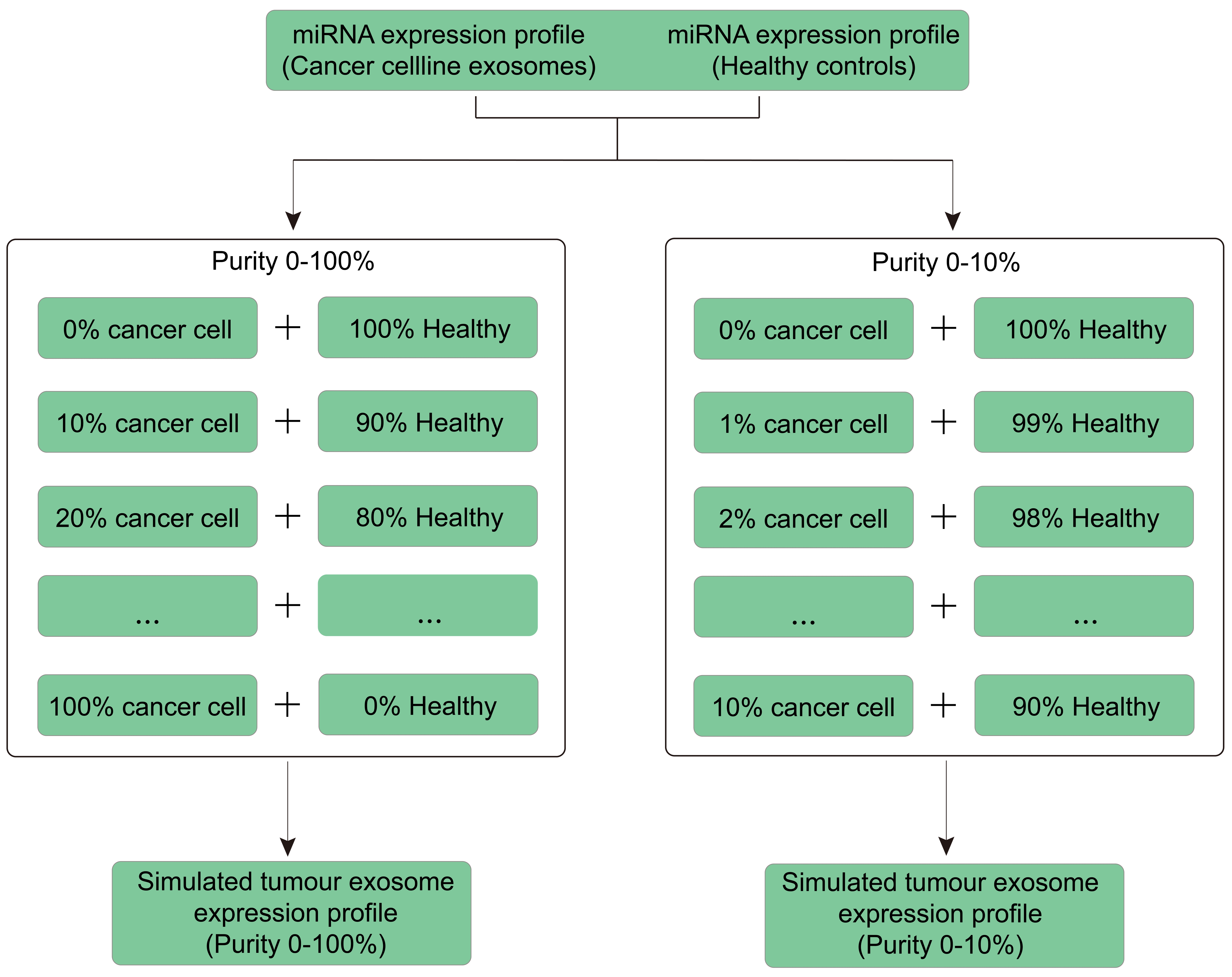
**

**Supplementary Figure S8. The process of generating simulated exosome miRNA-Seq data.** Simulated tumour exosome data with purity x% are designed by x% expression profile of cancer cell line and (100-x)% of healthy controls. Two purity ranges of datasets are simulated: (1) from 0 to 1 and (2) from 0 to 0.1.
