# Supplementary material for: ExosomePurity: tumour purity deconvolution in serum exosomes based on miRNA signatures
Source: Brief Bioinform. 2023 Mar 24;24(3):bbad119. doi: 10.1093/bib/bbad119 (PMC10199770; doi:10.1093/bib/bbad119)
Supplement: Table_S1_S2_S3_bbad119 [file table_s1_s2_s3_bbad119.docx]

**Supplementary Tables**

**Supplementary Table S1. The exosome miRNA-Seq datasets in the GEO database**

| **GEO accession number** | **Status** | **Samples** | **Bio Project** |
| --- | --- | --- | --- |
| GSE67004 | Colorectal cancer | 9 | PRJNA278673 |
| GSE125905 | Colorectal cancer | 12 | PRJNA517926 |
| GSE148698 | Colorectal cancer | 4 | PRJNA625457 |
| GSE125905 | Glioblastoma | 7 | PRJNA517926 |
| GSE138983 | Pancreatic cancer | 4 | PRJNA577933 |
| GSE109356 | Prostate cancer | 12 | PRJNA430677 |
| GSE106304 | Lung cancer | 8 | PRJNA416251 |
| GSE71895 | Breast cancer | 4 | PRJNA292422 |
| GSE83669 | Breast cancer | 3 | PRJNA326670 |
| GSE198354 | Gastric cancer | 6 | PRJNA814676 |
| GSE114920 | Ovarian cancer | 36 | PRJNA473131 |
| GSE161088 | Oesophageal cancer | 24 | PRJNA675537 |
| GSE128803 | Cervical cancer | 6 | PRJNA528920 |
| GSE158659 | Cervical cancer | 7 | PRJNA666144 |
| GSE84306 | Head and neck cancer | 12 | PRJNA328779 |
| GSE158659 | Head and neck cancer | 7 | PRJNA666144 |
| GSE128359 | Healthy control | 31 | PRJNA527257 |

**Supplementary Table S2. The external cohort of exosome miRNA-Seq datasets in the GEO database**

| **GEO accession number** | **Status** | **Samples** | **Bio Project** |
| --- | --- | --- | --- |
| GSE141326 | Breast cancer and healthy control | 4 | PRJNA593193 |
| GSE71008 | Colorectal cancer and healthy control | 38 | PRJNA290097 |
| GSE130654 | Gastric cancer and healthy control | 10 | PRJNA540915 |
| GSE122488 | Glioblastoma and healthy control | 20 | PRJNA505342 |
| GSE111803 | Lung cancer and healthy control | 4 | PRJNA438213 |
| GSE71008 | Pancreatic cancer and healthy control | 6 | PRJNA290097 |
| GSE71008 | Prostate cancer and healthy control | 10 | PRJNA290097 |
| GSE114711 | Lung cancer | 19 | PRJNA472351 |
| GSE71661 | Lung cancer and granuloma | 13 | PRJNA291756 |
| GSE160614 | Oesophageal cancer | 18 | PRJNA673798 |
| GSE158317 | Ovarian cancer | 3 | PRJNA664801 |
| GSE166799 | Pancreatic cancer | 2 | PRJNA701976 |
| GSE134205 | Prostate cancer | 6 | PRJNA554311 |
| GSE58410 | Prostate cancer | 10 | PRJNA252516 |
| GSE100467 | Healthy control | 34 | PRJNA391912 |
| GSE114711 | Healthy control | 7 | PRJNA472351 |
| GSE122621 | Healthy control | 4 | PRJNA505788 |
| GSE130654 | Healthy control | 12 | PRJNA540915 |
| GSE136997 | Healthy control | 12 | PRJNA564240 |
| GSE142819 | Healthy control | 15 | PRJNA598459 |
| GSE144781 | Healthy control | 5 | PRJNA604840 |
| GSE179382 | Healthy control | 8 | PRJNA743520 |

**Supplementary Table S3. The signatures in cancer exosomes**

| **Status** | **Nmuber** | **Signature** |
| --- | --- | --- |
| Breast cancer | 49 | hsa-miR-8068,hsa-miR-4511,hsa-miR-200c-3p,hsa-miR-148a-3p,hsa-miR-9-5p,hsa-miR-181c-5p,hsa-miR-6775-5p,hsa-miR-3132,hsa-miR-136-5p,hsa-miR-8088,hsa-miR-6846-3p,hsa-miR-4330,hsa-miR-95-3p,hsa-miR-6880-3p,hsa-miR-7111-5p,hsa-miR-4530,hsa-miR-6838-3p,hsa-miR-5196-3p,hsa-miR-221-5p,hsa-miR-4752,hsa-miR-3680-5p,hsa-miR-150-3p,hsa-miR-3670,hsa-miR-548w,hsa-miR-4517,hsa-miR-4294,hsa-miR-548t-3p,hsa-miR-6792-5p,hsa-miR-1178-3p,hsa-miR-4421,hsa-miR-3185,hsa-miR-12129,hsa-miR-1233-5p,hsa-miR-6728-3p,hsa-miR-6856-3p,hsa-miR-4718,hsa-miR-518a-3p,hsa-miR-135a-5p,hsa-miR-6762-3p,hsa-miR-4469,hsa-miR-3973,hsa-miR-95-5p,hsa-miR-6835-3p,hsa-miR-6880-5p,hsa-miR-3619-3p,hsa-miR-124-5p,hsa-miR-8055,hsa-miR-4454,hsa-miR-101-3p |
| Cervical cancer | 50 | hsa-miR-8068,hsa-miR-4511,hsa-miR-6784-5p,hsa-miR-148a-3p,hsa-miR-10398-5p,hsa-miR-3198,hsa-miR-4672,hsa-miR-217-5p,hsa-miR-4513,hsa-miR-181c-5p,hsa-miR-136-5p,hsa-miR-6716-5p,hsa-miR-8088,hsa-miR-6846-3p,hsa-miR-151b,hsa-miR-95-3p,hsa-miR-6880-3p,hsa-miR-7111-5p,hsa-miR-4530,hsa-miR-6838-3p,hsa-miR-5196-3p,hsa-miR-6829-3p,hsa-miR-221-5p,hsa-miR-4650-3p,hsa-miR-6781-5p,hsa-miR-4752,hsa-miR-7151-5p,hsa-miR-3670,hsa-miR-548w,hsa-miR-4517,hsa-miR-548t-3p,hsa-miR-12129,hsa-miR-6728-3p,hsa-miR-6856-3p,hsa-miR-518a-3p,hsa-miR-4706,hsa-miR-135a-5p,hsa-miR-6794-3p,hsa-miR-671-3p,hsa-miR-3179,hsa-miR-6511a-5p,hsa-miR-6864-3p,hsa-miR-6770-5p,hsa-miR-6893-3p,hsa-miR-95-5p,hsa-miR-6835-3p,hsa-miR-124-5p,hsa-miR-8055,hsa-miR-4454,hsa-miR-101-3p |
| Colorectal cancer | 48 | hsa-miR-8068,hsa-miR-4511,hsa-miR-200c-3p,hsa-miR-148a-3p,hsa-miR-217-5p,hsa-miR-181c-5p,hsa-miR-6775-5p,hsa-miR-136-5p,hsa-miR-8088,hsa-miR-6846-3p,hsa-miR-4330,hsa-miR-151b,hsa-miR-95-3p,hsa-miR-6880-3p,hsa-miR-7111-5p,hsa-miR-4530,hsa-miR-6838-3p,hsa-miR-5196-3p,hsa-miR-6829-3p,hsa-miR-221-5p,hsa-miR-1273c,hsa-miR-4752,hsa-miR-3680-5p,hsa-miR-150-3p,hsa-miR-3670,hsa-miR-548w,hsa-miR-4517,hsa-miR-548t-3p,hsa-miR-6792-5p,hsa-miR-1178-3p,hsa-miR-184,hsa-miR-4421,hsa-miR-3185,hsa-miR-29b-1-5p,hsa-miR-12129,hsa-miR-6728-3p,hsa-miR-6856-3p,hsa-miR-518a-3p,hsa-miR-135a-5p,hsa-miR-3179,hsa-miR-6864-3p,hsa-miR-6770-5p,hsa-miR-6893-3p,hsa-miR-95-5p,hsa-miR-3619-3p,hsa-miR-124-5p,hsa-miR-8055,hsa-miR-4454 |
| Gastric cancer | 52 | hsa-miR-8068,hsa-miR-4511,hsa-miR-483-5p,hsa-miR-200c-3p,hsa-miR-217-5p,hsa-miR-181c-5p,hsa-miR-6775-5p,hsa-miR-3132,hsa-miR-136-5p,hsa-miR-8088,hsa-miR-6846-3p,hsa-miR-4330,hsa-miR-95-3p,hsa-miR-6880-3p,hsa-miR-7111-5p,hsa-miR-1233-3p,hsa-miR-4530,hsa-miR-6838-3p,hsa-miR-5196-3p,hsa-miR-6829-3p,hsa-miR-221-5p,hsa-miR-1273c,hsa-miR-4752,hsa-miR-3680-5p,hsa-miR-7151-5p,hsa-miR-150-3p,hsa-miR-3670,hsa-miR-92b-5p,hsa-miR-548w,hsa-miR-4517,hsa-miR-548t-3p,hsa-miR-6792-5p,hsa-miR-1178-3p,hsa-miR-184,hsa-miR-4421,hsa-miR-3185,hsa-miR-4642,hsa-miR-29b-1-5p,hsa-miR-761,hsa-miR-12129,hsa-miR-6728-3p,hsa-miR-6856-3p,hsa-miR-518a-3p,hsa-miR-135a-5p,hsa-miR-5091,hsa-miR-6864-3p,hsa-miR-6770-5p,hsa-miR-95-5p,hsa-miR-6880-5p,hsa-miR-6890-5p,hsa-miR-3619-3p,hsa-miR-4518 |
| Glioblastoma | 76 | hsa-miR-483-5p,hsa-miR-200c-3p,hsa-miR-6865-3p,hsa-miR-6775-3p,hsa-miR-3173-5p,hsa-miR-518e-5p,hsa-let-7f-2-3p,hsa-miR-487b-3p,hsa-miR-3187-3p,hsa-miR-217-5p,hsa-miR-181c-5p,hsa-miR-6775-5p,hsa-miR-3132,hsa-miR-136-5p,hsa-miR-210-3p,hsa-miR-8088,hsa-miR-6846-3p,hsa-miR-4330,hsa-miR-95-3p,hsa-miR-6880-3p,hsa-miR-7111-5p,hsa-miR-1233-3p,hsa-miR-4530,hsa-miR-6838-3p,hsa-miR-5196-3p,hsa-miR-221-5p,hsa-miR-7-5p,hsa-miR-1273c,hsa-miR-4752,hsa-miR-3680-5p,hsa-miR-150-3p,hsa-miR-6866-3p,hsa-miR-3670,hsa-miR-92b-5p,hsa-miR-548w,hsa-miR-4517,hsa-miR-548t-3p,hsa-miR-6792-5p,hsa-miR-1178-3p,hsa-miR-184,hsa-miR-4421,hsa-miR-3185,hsa-miR-503-5p,hsa-miR-4642,hsa-miR-29b-1-5p,hsa-miR-761,hsa-miR-12129,hsa-miR-6728-3p,hsa-miR-6856-3p,hsa-miR-518a-3p,hsa-miR-135a-5p,hsa-miR-3179,hsa-miR-6864-3p,hsa-miR-6770-5p,hsa-miR-626,hsa-miR-95-5p,hsa-miR-518e-3p,hsa-miR-4504,hsa-miR-4468,hsa-miR-135a-3p,hsa-miR-571,hsa-miR-6880-5p,hsa-miR-125a-5p,hsa-miR-6890-5p,hsa-miR-3680-3p,hsa-miR-5088-5p,hsa-miR-3619-3p,hsa-miR-4518,hsa-miR-483-3p,hsa-miR-3945,hsa-miR-124-5p,hsa-miR-192-3p,hsa-miR-518a-5p,hsa-miR-6808-3p,hsa-miR-8055,hsa-miR-101-3p |
| Head and neck cancer | 25 | hsa-miR-8068,hsa-miR-4511,hsa-miR-10398-5p,hsa-miR-217-5p,hsa-miR-136-5p,hsa-miR-7855-5p,hsa-miR-4330,hsa-miR-151b,hsa-miR-4530,hsa-miR-6838-3p,hsa-miR-221-5p,hsa-miR-7151-5p,hsa-miR-12122,hsa-miR-150-3p,hsa-miR-3670,hsa-miR-92b-5p,hsa-miR-4517,hsa-miR-548t-3p,hsa-miR-12129,hsa-miR-6770-5p,hsa-miR-6893-3p,hsa-miR-95-5p,hsa-miR-124-5p,hsa-miR-8055,hsa-miR-4454 |
| Lung cancer | 63 | hsa-miR-483-5p,hsa-miR-200c-3p,hsa-let-7f-2-3p,hsa-miR-378j,hsa-miR-487b-3p,hsa-miR-217-5p,hsa-miR-6775-5p,hsa-miR-3132,hsa-miR-7855-5p,hsa-miR-210-3p,hsa-miR-8088,hsa-miR-5193,hsa-miR-4787-3p,hsa-miR-6846-3p,hsa-miR-4330,hsa-miR-95-3p,hsa-miR-6880-3p,hsa-miR-194-5p,hsa-miR-1233-3p,hsa-miR-4530,hsa-miR-6838-3p,hsa-miR-5196-3p,hsa-miR-221-5p,hsa-miR-7-5p,hsa-miR-1273c,hsa-miR-4752,hsa-miR-3680-5p,hsa-miR-7151-5p,hsa-miR-12122,hsa-miR-150-3p,hsa-miR-3670,hsa-miR-92b-5p,hsa-miR-548w,hsa-miR-4517,hsa-miR-1258,hsa-miR-6731-5p,hsa-miR-548t-3p,hsa-miR-6792-5p,hsa-miR-1178-3p,hsa-miR-184,hsa-miR-4421,hsa-miR-503-5p,hsa-miR-4642,hsa-miR-761,hsa-miR-12129,hsa-miR-6728-3p,hsa-miR-6856-3p,hsa-miR-4718,hsa-miR-518a-3p,hsa-miR-135a-5p,hsa-miR-6864-3p,hsa-miR-626,hsa-miR-6893-3p,hsa-miR-95-5p,hsa-miR-518e-3p,hsa-miR-4504,hsa-miR-3680-3p,hsa-miR-5088-5p,hsa-miR-124-5p,hsa-miR-6808-3p,hsa-miR-8055,hsa-miR-4454,hsa-miR-101-3p |
| Oesophageal cancer | 63 | hsa-miR-8068,hsa-miR-4511,hsa-miR-200c-3p,hsa-miR-5004-5p,hsa-miR-148a-3p,hsa-miR-518e-5p,hsa-miR-615-3p,hsa-miR-487b-3p,hsa-miR-10398-5p,hsa-miR-3198,hsa-miR-3187-3p,hsa-miR-217-5p,hsa-miR-4513,hsa-miR-100-3p,hsa-miR-181c-5p,hsa-miR-6775-5p,hsa-miR-136-5p,hsa-miR-6716-5p,hsa-miR-210-3p,hsa-miR-4787-3p,hsa-miR-6846-3p,hsa-miR-4330,hsa-miR-1233-3p,hsa-miR-4530,hsa-miR-5196-3p,hsa-miR-221-5p,hsa-miR-7-5p,hsa-miR-1273c,hsa-miR-4752,hsa-miR-3680-5p,hsa-miR-12122,hsa-miR-150-3p,hsa-miR-92b-5p,hsa-miR-548w,hsa-miR-4517,hsa-miR-548t-3p,hsa-miR-6792-5p,hsa-miR-1178-3p,hsa-miR-184,hsa-miR-4421,hsa-miR-3185,hsa-miR-29b-1-5p,hsa-miR-761,hsa-miR-12129,hsa-miR-6728-3p,hsa-miR-6856-3p,hsa-miR-518a-3p,hsa-miR-135a-5p,hsa-miR-6794-3p,hsa-miR-3179,hsa-miR-6864-3p,hsa-miR-6770-5p,hsa-miR-6893-3p,hsa-miR-95-5p,hsa-miR-571,hsa-miR-6880-5p,hsa-miR-6890-5p,hsa-miR-5088-5p,hsa-miR-3619-3p,hsa-miR-124-5p,hsa-miR-8055,hsa-miR-4454,hsa-miR-101-3p |
| Ovarian cancer | 22 | hsa-miR-518e-5p,hsa-let-7f-2-3p,hsa-miR-6775-5p,hsa-miR-6862-5p,hsa-miR-151b,hsa-miR-95-3p,hsa-miR-4530,hsa-miR-5196-3p,hsa-miR-221-5p,hsa-miR-503-5p,hsa-miR-6728-3p,hsa-miR-518a-3p,hsa-miR-135a-5p,hsa-miR-3179,hsa-miR-6762-3p,hsa-miR-4469,hsa-miR-6770-5p,hsa-miR-3973,hsa-miR-6893-3p,hsa-miR-4771,hsa-miR-4468,hsa-miR-571 |
| Pancreatic cancer | 33 | hsa-miR-7844-5p,hsa-miR-9-5p,hsa-miR-518e-5p,hsa-miR-6510-3p,hsa-miR-217-5p,hsa-miR-6775-5p,hsa-miR-7855-5p,hsa-miR-210-3p,hsa-miR-6846-3p,hsa-miR-4330,hsa-miR-4530,hsa-miR-6838-3p,hsa-miR-1273c,hsa-miR-3680-5p,hsa-miR-12122,hsa-miR-150-3p,hsa-miR-92b-5p,hsa-miR-498-3p,hsa-miR-548w,hsa-miR-4517,hsa-miR-548t-3p,hsa-miR-6792-5p,hsa-miR-1178-3p,hsa-miR-184,hsa-miR-4421,hsa-miR-12129,hsa-miR-6728-3p,hsa-miR-6856-3p,hsa-miR-518a-3p,hsa-miR-135a-5p,hsa-miR-6893-3p,hsa-miR-95-5p,hsa-miR-101-3p |
| Prostate cancer | 67 | hsa-miR-8068,hsa-miR-4511,hsa-miR-483-5p,hsa-miR-200c-3p,hsa-miR-148a-3p,hsa-miR-518e-5p,hsa-miR-615-3p,hsa-miR-10398-5p,hsa-miR-3198,hsa-miR-4672,hsa-miR-217-5p,hsa-miR-4513,hsa-miR-100-3p,hsa-miR-181c-5p,hsa-miR-6775-5p,hsa-miR-3132,hsa-miR-136-5p,hsa-miR-6716-5p,hsa-miR-210-3p,hsa-miR-6846-3p,hsa-miR-4330,hsa-miR-151b,hsa-miR-4746-5p,hsa-miR-95-3p,hsa-miR-7111-5p,hsa-miR-1233-3p,hsa-miR-4530,hsa-miR-5196-3p,hsa-miR-6829-3p,hsa-miR-221-5p,hsa-miR-4650-3p,hsa-miR-1273c,hsa-miR-3680-5p,hsa-miR-150-3p,hsa-miR-3670,hsa-miR-92b-5p,hsa-miR-548w,hsa-miR-4517,hsa-miR-548t-3p,hsa-miR-6792-5p,hsa-miR-3180-5p,hsa-miR-1178-3p,hsa-miR-184,hsa-miR-4421,hsa-miR-3185,hsa-miR-29b-1-5p,hsa-miR-761,hsa-miR-12129,hsa-miR-6728-3p,hsa-miR-6856-3p,hsa-miR-518a-3p,hsa-miR-4706,hsa-miR-135a-5p,hsa-miR-6794-3p,hsa-miR-671-3p,hsa-miR-3179,hsa-miR-6864-3p,hsa-miR-6770-5p,hsa-miR-6893-3p,hsa-miR-95-5p,hsa-miR-571,hsa-miR-6880-5p,hsa-miR-6890-5p,hsa-miR-5088-5p,hsa-miR-3619-3p,hsa-miR-4518,hsa-miR-101-3p |
| Pan-cancer | 46 | hsa-miR-4530,hsa-miR-12129,hsa-miR-135a-5p,hsa-miR-221-5p,hsa-miR-4517,hsa-miR-518a-3p,hsa-miR-548t-3p,hsa-miR-6728-3p,hsa-miR-95-5p,hsa-miR-150-3p,hsa-miR-217-5p,hsa-miR-4330,hsa-miR-5196-3p,hsa-miR-548w,hsa-miR-6775-5p,hsa-miR-6846-3p,hsa-miR-6856-3p,hsa-miR-1178-3p,hsa-miR-136-5p,hsa-miR-3670,hsa-miR-3680-5p,hsa-miR-4421,hsa-miR-6770-5p,hsa-miR-6792-5p,hsa-miR-6838-3p,hsa-miR-6893-3p,hsa-miR-95-3p,hsa-miR-101-3p,hsa-miR-124-5p,hsa-miR-1273c,hsa-miR-181c-5p,hsa-miR-184,hsa-miR-200c-3p,hsa-miR-4511,hsa-miR-4752,hsa-miR-6864-3p,hsa-miR-8055,hsa-miR-8068,hsa-miR-92b-5p,hsa-miR-3179,hsa-miR-3185,hsa-miR-3619-3p,hsa-miR-4454,hsa-miR-6880-3p,hsa-miR-7111-5p,hsa-miR-8088 |
